# A stochastic fractional model for computer virus propagation with memory effects and exponential delay

**DOI:** 10.1371/journal.pone.0360254

**Published:** 2026-10-14

**Authors:** Ali Raza, Marek Lampart, Umar Shafique, Dumitru Baleanu, Maysaa Elmahi Abd Elwahab

**Affiliations:** 1 Department of Mathematics, Fırat University, Elazığ, Türkiye; 2 IT4Innovations, VSB–Technical University of Ostrava, Ostrava, Czech Republic; 3 Faculty of Electrical Engineering and Computer Science, VSB–Technical University of Ostrava, Ostrava, Czech Republic; 4 Department of Computer Science and Mathematics, Lebanese American University, Beirut, Lebanon; 5 Department of Mathematical Sciences, College of Science, Princess Nourah bint Abdulrahman University, Riyadh, Saudi Arabia; Lanzhou University of Technology, CHINA

## Abstract

A computer virus is a type of malware that, upon execution, can replicate itself by modifying other computer programs and inserting its code into them. This study develops a stochastic fractional-order delay differential equation model (SFDDE) to describe computer-virus dynamics by partitioning the system into uninfected *S*(*t*), latent infected *L*(*t*), and actively seizing *V*(*t*) computer populations. The model establishes the well-posedness of solutions, including existence, uniqueness, positivity, and boundedness, and identifies two equilibria: the computer-virus-free equilibrium (CV-FE) and the computer-virus-present equilibrium (CV-PE). The basic reproduction number is derived using the next-generation matrix method, followed by local and global stability analyses of the equilibrium points. A sensitivity analysis is performed to assess the influence of key parameters on virus transmission. Furthermore, a stochastic Non-Standard Finite Difference (NSFD) scheme is constructed to preserve the essential qualitative properties of the model, and its numerical performance is compared with the fractional Euler–Maruyama method using step-size refinement and quantitative error measures. The numerical experiments illustrate the effects of fractional memory, transmission delay, and stochastic perturbations on computer-virus dynamics. The results provide mathematical insight into computer-virus propagation and indicate the importance of reducing virus transmission and improving detection, removal, and recovery processes for effective cybersecurity management. The study demonstrates the usefulness of stochastic fractional-order delay modeling and structure-preserving numerical methods for analyzing complex computer-virus dynamics.

## 1 Introduction

In [1], the authors developed and critically analyzed NSFD schemes tailored for a computer virus propagation. The schemes preserve the system’s qualitative dynamics, thereby accurately representing the spread of the virus over time. In [2], the authors consider constrained resource availability as it impacts how to develop proper strategies for computer virus prevention and control. That is, there is a balancing of protection with system performance. In [3], the authors explored and extended the use of advanced neural networks, like Graph Neural Networks, to computer virus spread models and analysis over diverse systems so that they obtain better accuracy levels, higher rates of convergence, and lesser computation complexity. In [4], the authors analyzed the dynamics of computer viruses using a fractional epidemiological model, which is formulated using the Caputo derivative. An advanced computational approach, the Jacobi collocation operational matrix method, is employed to solve the fractional-order model with high accuracy and efficiency for the numerical solutions. In [5], the authors used the wavelet method due to Fibonacci as an innovative approach to finding approximate solutions for the nonlinear modified epidemiological model of computer viruses. A method based on the operational matrix of integration (OMI) constructed via Fibonacci polynomials enhances computational accuracy and efficiency in the solution processes. In [6], the authors have considered the application of the epidemiological model in the study of computer viruses to identify dynamic multiple virus transmission mechanisms in digital ecosystems. It would thus help provide a strategy for tools intended for controlling, countering, or restriction the spread of such viruses and reducing further damage due to these viruses. In [7], the authors have discussed dynamic analysis of the propagation of computer viruses in point-to-group information networks, taking into account the urgent challenges such threats pose for the modern digital ecosystem. The paper further considers optimal control strategies to counteract the spread and impact of computer viruses, hence improving network security and resilience. In [8], the authors employed the Caputo fractional operator to derive valid and reliable results. This paper formulates the conjunctivitis virus transmission model in fractional order and establishes the same. The strategy focuses on early detection and immunization among vulnerable populations with a weak immune system for enhanced prevention and control. In [9], the authors proposed a novel and robust control strategy that overcomes the spread of computer viruses based on the optimal control strategy. This research methodology, through the SISRS model, challenges and addresses the main issue with the diffusion of viruses in computing environments to build a bright approach toward reducing cyber threats’ adverse impacts. A model of the spread of computer viruses using fuzzy parameters is proposed by the authors in [10]. The model aims to make virus propagation dynamics more understandable. Introducing fuzzy logic makes the uncertainty and variability present in the most significant factors that may affect the spreading of the virus clear, making the model even more predictive. In [11], authors have numerically intensive stochastic methods that solve a family of mathematical models of the fractional order that model computer virus propagation with kill signals. The detail is about researching the kill signal model in computer virus propagation and making use of fractional derivatives to make a deeper analysis of the dynamics that characterize virus spread and kill signals’ effect in computational systems. In [12], the authors presented several optimal control techniques that try to minimize the spreading of the virus in computer networks. The strategies proposed here try to minimize the damage imposed by the spreading of viruses on computers while simultaneously decreasing the costs of installing and maintaining anti-virus software. In [13], the authors studied the optimal control analysis of a computer virus model wherein we used the Atangana-Baleanu derivative to include fractional dynamics. We then applied a fixed-point theorem to study the solutions of this fractional-order model so that we may rigorously ensure a mathematical framework for solution existence and stability. In [14], the authors investigate a mathematical model that discusses the dynamic behavior and impacts of the Stuxnet virus on computer systems. The study looks into the structural effects that the virus roots its influence in; it clarifies how these spreads and interacts with the components of the system. In [15], the authors presented a deep analytical and numerical analysis of a computer virus epidemic model that was motivated by the complicated dynamics of the virus spread. For better investigation of the system behavior, the conventional model was modified into a fractional order model to further understand the dynamics. In [16], the mechanism of infection with a computer virus was illustrated while explaining how this might spread into and across a vulnerable system. Next, we proceed to demonstrate the antidote through which it would be introduced and counteract nullifying the spreading of the virus, thereby reintroducing integrity back into the system. In [17], authors have used the fractal-fractional derivative operator in the context of investigating how memory effects may impact the population dynamics in computer viruses. Analysis has been proposed to enhance understanding of the behavior complexity and long-time evolution of populations of computer viruses in light of memory-dependent interactions and patterns. In [18], the authors investigated a fractional-order SEIR-KS model that uses the Caputo derivative to discuss the role of nodes that act as kill signals in forming the dynamics of virus spread. Here, our goal is to observe if these nodes have a crucial role to play in affecting the infection-spreading process as well as in mechanisms controlling the infection. The authors, in [19], modified the Susceptible-Infected-Removed (SIR) epidemiological model by further incorporating an antidotal population to better give the real-world situations. They applied the fractional natural decomposition method along with the variational iteration method to analyze and solve the governing equations to understand the behavioral dynamics of the modified model. In [20], the authors proposed a new fractional numerical algorithm for an accurate solution to generalized fractional order differential equations in the Caputo sense with order such that 0<β<1. The basic idea of this new method is to find the exact solution for the wide variety of fractional models, so one may improve computational efficiency and reliability while solving those equations [21]. The proposed numerical treatment is consistent with recent developments in fractional-order and delayed fractional systems [22–24].

The main contributions of this work are summarized as follows:

- We formulate a stochastic fractional-order computer-virus model incorporating both time-delay effects and stochastic perturbations, providing a unified framework for describing memory, delayed transmission, and random fluctuations in computer-virus dynamics.
- We develop a stochastic nonstandard finite difference (NSFD) discretization for the proposed fractional-order delayed system, with emphasis on obtaining controlled numerical behavior and preserving the qualitative features of the model.
- We investigate numerically the effects of the fractional order, time delay, and stochastic intensity on the evolution of the uninfected, latent, and seizing computer populations.
- We compare the proposed stochastic NSFD method with the fractional Euler–Maruyama method using trajectory behavior, numerical error, and time-step sensitivity, thereby demonstrating the computational characteristics of the proposed discretization.
- We further compare deterministic and stochastic dynamics to illustrate the effect of random perturbations on the computer-virus propagation process.

In this paper, Section 1 reviews computer virus literature, including epidemiology, lifecycle mechanisms, and modeling gaps. In Section 2, a stochastic fractional model is developed and some of its important properties are analyzed. Section 3 presents the stochastic NSFD scheme and compares its positivity, boundedness, and stability. Section 4 provides simulations and discusses their practical implications. Section 5 summarizes the findings and suggests future research directions.

## 2 Model formulation and deterministic analytical framework

We consider a computer-virus propagation model in which computers connected to the Internet are classified into three compartments: uninfected computers *S*(*t*), latent computers *L*(*t*), and seizing (compromised) computers *V*(*t*). The total normalized computer population is assumed to satisfy


$$\begin{array}{c} S(t)+L(t)+V(t)=1. \end{array}$$

New internal computers enter the system at rate Λ and leave the system at rate δ, and all newly introduced computers are assumed to be virus-free. Since both latent and seizing computers are considered infectious, virus transmission occurs at the delayed incidence rate


$$\begin{array}{c} \beta S(t-\tau )[L(t-\tau )+V(t-\tau )], \end{array}$$

where β is the transmission rate and τ≥0 is the time delay. Latent computers become seizing at rate θ, whereas latent and seizing computers are cured at rates $\gamma_{1}$ and $\gamma_{2}$, respectively, with $\gamma_{2}>\gamma_{1}>0$.

Following the computer-virus model in [1], the classical first-order temporal derivatives are replaced by Caputo fractional derivatives of order α, where 0<α≤1. The Caputo derivative incorporates memory effects by weighting the previous history of the system in the evolution of the current state. For a sufficiently smooth function *f*(*t*) and 0<α<1, it is defined by


$$\begin{array}{c} {\;}_{0}^{C}D_{t}^{\alpha }f(t)=\frac{1}{\Gamma (1-\alpha )}\int_{0}^{t}\frac{f^{\prime }(\xi )}{(t-\xi {)}^{\alpha }}\;d\xi , \end{array}$$ (1)

where Γ(·) denotes the Gamma function. For α=1, the Caputo derivative reduces to the classical first-order derivative, that is,


$$\begin{array}{c} {\;}_{0}^{C}D_{t}^{1}f(t)=\frac{df(t)}{dt}. \end{array}$$

The use of the Caputo derivative is appropriate for the present application because computer-virus propagation may depend on the previous infection, latency, recovery, and compromise history of the computer population. Thus, the fractional order provides a mathematical mechanism for incorporating memory effects into the virus-propagation dynamics.

With the above assumptions, the proposed stochastic fractional-order delay model is given by


$$\begin{array}{c} {\;}_{0}^{C}D_{t}^{\alpha }S(t)=\Lambda^{\alpha }-\beta^{\alpha }S(t-\tau )\left[L(t-\tau )+V(t-\tau )\right]e^{-\delta^{\alpha }\tau }-\delta^{\alpha }S(t)+\gamma_{1}^{\alpha }L(t)+\gamma_{2}^{\alpha }V(t)+\sigma_{1}S(t)\;dB(t), \end{array}$$ (2)

$$\begin{array}{c} {\;}_{0}^{C}D_{t}^{\alpha }L(t)=\beta^{\alpha }S(t-\tau )\left[L(t-\tau )+V(t-\tau )\right]e^{-\delta^{\alpha }\tau }-\left(\gamma_{1}^{\alpha }+\theta^{\alpha }+\delta^{\alpha }\right)L(t)+\sigma_{2}L(t)\;dB(t), \end{array}$$ (3)

$$\begin{array}{c} {\;}_{0}^{C}D_{t}^{\alpha }V(t)=\theta^{\alpha }L(t)-\left(\gamma_{2}^{\alpha }+\delta^{\alpha }\right)V(t)+\sigma_{3}V(t)\;dB(t). \end{array}$$ (4)

Here, *S*(*t*), *L*(*t*), and *V*(*t*) denote the uninfected, latent, and seizing computer populations, respectively. The parameters Λ, β, and δ represent the recruitment, transmission, and removal rates, respectively. The parameters $\gamma_{1}$ and $\gamma_{2}$ denote the curing rates of latent and seizing computers, while θ represents the transition rate from the latent to the seizing class. The parameter τ is the nonnegative delay associated with the delayed infection process, and α∈(0,1] is the fractional order.

The stochastic perturbations are represented by $\sigma_{1}S(t)\;dB(t)$, $\sigma_{2}L(t)\;dB(t)$, and $\sigma_{3}V(t)\;dB(t)$, where $\sigma_{i}\geq 0$, *i* = 1,2,3, are the stochastic intensity parameters and *B*(*t*) is a standard Brownian motion. The stochastic terms are assumed to be adapted to the underlying filtration and to satisfy the regularity condi*t*ions required for the stochastic formulation and numerical scheme. The parameters $\sigma_{i}$ determine the magnitude of the random perturbations acting on the corresponding computer populations.

The system is subject to the nonnegative initial conditions


$$\begin{array}{c} S(0)\geq 0,\;L(0)\geq 0,\;V(0)\geq 0, \end{array}$$ (5)

together with prescribed history functions *S*(*t*), *L*(*t*), and *V*(*t*) on [−τ,0] when τ>0.

The time-delay parameter τ represents a deterministic time lag in the infection mechanism and is distinct from the stochastic perturbations governed by the Brownian motion. When $\sigma_{1}=\sigma_{2}=\sigma_{3}=0$, system (2)–(4) reduces to the corresponding deterministic fractional-order delay system. The analytical results in Sections 2.1–2.5 are established for this deterministic system. The stochastic model is subsequently investigated numerically using the proposed stochastic NSFD scheme.

### 2.1 Existence and uniqueness

For the theoretical analysis, we consider the deterministic fractional-order counterpart of system (2)–(4), obtained by setting $\sigma_{i}=0$, *i* = 1,2,3. Let


$$\begin{array}{c} X(t)=(S(t),L(t),V(t){)}^{T}. \end{array}$$

Then system (2)–(4) can be written in the compact form


$$\begin{array}{c} {\;}_{0}^{C}D_{t}^{\alpha }X(t)=F(X(t)),\;0<\alpha <1, \end{array}$$

where


$$\begin{array}{c} F(X)=(\begin{array}{c} \hbar_{1}(S,L,V)\\ \hbar_{2}(S,L,V)\\ \hbar_{3}(S,L,V) \end{array}). \end{array}$$

The component functions are


$$\begin{array}{c} \hbar_{1}(S,L,V)=\Lambda^{\alpha }-\beta^{\alpha }S(L+V)e^{-\delta^{\alpha }\tau }-\delta^{\alpha }S+\gamma_{1}^{\alpha }L+\gamma_{2}^{\alpha }V. \end{array}$$ (6)

$$\begin{array}{c} \hbar_{2}(S,L,V)=\beta^{\alpha }S(L+V)e^{-\delta^{\alpha }\tau }-\left(\gamma_{1}^{\alpha }+\theta^{\alpha }+\delta^{\alpha }\right)L. \end{array}$$ (7)

$$\begin{array}{c} \hbar_{3}(S,L,V)=\theta^{\alpha }L-\left(\gamma_{2}^{\alpha }+\delta^{\alpha }\right)V. \end{array}$$ (8)

Let the initial conditions be


$$\begin{array}{c} S(0)=S_{0},\;L(0)=L_{0},\;V(0)=V_{0}, \end{array}$$

with $S_{0},L_{0},V_{0}\geq 0$. Since the right-hand side is continuous, applying the fractional integral operator to the Caputo system gives the equivalent Volterra integral equations.


$$\begin{array}{c} S(t)=S_{0}+\frac{1}{\Gamma (\alpha )}\int_{0}^{t}(t-s{)}^{\alpha -1}\hbar_{1}(S(s),L(s),V(s))\;ds. \end{array}$$ (9)

$$\begin{array}{c} L(t)=L_{0}+\frac{1}{\Gamma (\alpha )}\int_{0}^{t}(t-s{)}^{\alpha -1}\hbar_{2}(S(s),L(s),V(s))\;ds. \end{array}$$ (10)

$$\begin{array}{c} V(t)=V_{0}+\frac{1}{\Gamma (\alpha )}\int_{0}^{t}(t-s{)}^{\alpha -1}\hbar_{3}(S(s),L(s),V(s))\;ds. \end{array}$$ (11)

We now establish existence and uniqueness using the Banach fixed-point theorem. Let


$$\begin{array}{c} 𝒳=C([0,T],{\mathbb{R}}^{3}) \end{array}$$

be the Banach space of continuous vector-valued functions equipped with the supremum norm


$$\begin{array}{c} \|X{\|}_{\infty }=\max_{0\leq t\leq T}\max\{|S(t)|,|L(t)|,|V(t)|\}. \end{array}$$

For a fixed *R* > 0, define the closed ball


$$\begin{array}{c} {ℬ}_{R}=\left\{X\in 𝒳:\|X-X_{0}{\|}_{\infty }\leq R\right\},\;X_{0}=(S_{0},L_{0},V_{0}{)}^{T}. \end{array}$$

Because 𝒳 is complete and ${ℬ}_{R}$ is a closed subset of 𝒳, ${ℬ}_{R}$ is itself a complete metric space. Define the operator


$$\begin{array}{c} 𝒯:{ℬ}_{R}⟶𝒳 \end{array}$$

by


$$\begin{array}{c} (𝒯X)(t)=X_{0}+\frac{1}{\Gamma (\alpha )}\int_{0}^{t}(t-s{)}^{\alpha -1}F(X(s))\;ds. \end{array}$$

We first establish the Lipschitz condition. Let


$$\begin{array}{c} X=(S,L,V{)}^{T},\;Y=(\tilde{S},\tilde{L},\tilde{V}{)}^{T}\in {ℬ}_{R}. \end{array}$$

Since ${ℬ}_{R}$ is bounded, there exists *M* > 0 such that


$$\begin{array}{c} |S|,|\tilde{S}|\leq M,\;|L|,|\tilde{L}|\leq M,\;|V|,|\tilde{V}|\leq M. \end{array}$$

For the nonlinear incidence term, we have


$$\begin{array}{c} \begin{array}{cc} & \left|S(L+V)-\tilde{S}(\tilde{L}+\tilde{V})\right|\\ & \;\leq |S-\tilde{S}|\;|L+V|+|\tilde{S}|\left(|L-\tilde{L}|+|V-\tilde{V}|\right)\\ & \;\leq 2M|S-\tilde{S}|+M|L-\tilde{L}|+M|V-\tilde{V}|. \end{array} \end{array}$$

Consequently,


$$\begin{array}{c} \begin{array}{cc} |\hbar_{1}(X)-\hbar_{1}(Y)|\leq & \left(2\beta^{\alpha }Me^{-\delta^{\alpha }\tau }+\delta^{\alpha }\right)|S-\tilde{S}|\\ & +\left(\beta^{\alpha }Me^{-\delta^{\alpha }\tau }+\gamma_{1}^{\alpha }\right)|L-\tilde{L}|\\ & +\left(\beta^{\alpha }Me^{-\delta^{\alpha }\tau }+\gamma_{2}^{\alpha }\right)|V-\tilde{V}|. \end{array} \end{array}$$

Similarly,


$$\begin{array}{c} \begin{array}{cc} |\hbar_{2}(X)-\hbar_{2}(Y)|\leq & \beta^{\alpha }Me^{-\delta^{\alpha }\tau }|S-\tilde{S}|\\ & +\left(\beta^{\alpha }Me^{-\delta^{\alpha }\tau }+\gamma_{1}^{\alpha }+\theta^{\alpha }+\delta^{\alpha }\right)|L-\tilde{L}|\\ & +\beta^{\alpha }Me^{-\delta^{\alpha }\tau }|V-\tilde{V}|. \end{array} \end{array}$$

$$\begin{array}{c} \begin{array}{cc} |\hbar_{3}(X)-\hbar_{3}(Y)|\leq & \theta^{\alpha }|L-\tilde{L}|\\ & +(\gamma_{2}^{\alpha }+\delta^{\alpha })|V-\tilde{V}|. \end{array} \end{array}$$

Define


$$\begin{array}{c} \begin{array}{cc} K_{1}= & 2\beta^{\alpha }Me^{-\delta^{\alpha }\tau }+\delta^{\alpha }+\beta^{\alpha }Me^{-\delta^{\alpha }\tau }+\gamma_{1}^{\alpha }\\ & +\beta^{\alpha }Me^{-\delta^{\alpha }\tau }+\gamma_{2}^{\alpha },\\ K_{2}= & \beta^{\alpha }Me^{-\delta^{\alpha }\tau }+\beta^{\alpha }Me^{-\delta^{\alpha }\tau }+\gamma_{1}^{\alpha }+\theta^{\alpha }+\delta^{\alpha }\\ & +\beta^{\alpha }Me^{-\delta^{\alpha }\tau },\\ K_{3}= & \theta^{\alpha }+\gamma_{2}^{\alpha }+\delta^{\alpha }, \end{array} \end{array}$$

and let


$$\begin{array}{c} K=\max\{K_{1},K_{2},K_{3}\}. \end{array}$$

Using the supremum norm, the preceding estimates imply


$$\begin{array}{c} \|F(X)-F(Y){\|}_{\infty }\leq K\|X-Y{\|}_{\infty }. \end{array}$$

Thus, *F* is locally Lipschitz continuous on ${ℬ}_{R}$. Next, for $X,Y\in {ℬ}_{R}$, the definition of 𝒯 and the Lipschitz estimate give


$$\begin{array}{c} \begin{array}{cc} \|𝒯X-𝒯Y{\|}_{\infty } & \leq \frac{K}{\Gamma (\alpha )}\sup_{0\leq t\leq T}\int_{0}^{t}(t-s{)}^{\alpha -1}\|X-Y{\|}_{\infty }\;ds\\ & =\frac{KT^{\alpha }}{\Gamma (\alpha +1)}\|X-Y{\|}_{\infty }. \end{array} \end{array}$$

Therefore, the contraction constant is


$$\begin{array}{c} q=\frac{KT^{\alpha }}{\Gamma (\alpha +1)}. \end{array}$$

Choose *T* > 0 sufficiently small such that


$$\begin{array}{c} q=\frac{KT^{\alpha }}{\Gamma (\alpha +1)}<1. \end{array}$$

It remains to verify that 𝒯 maps ${ℬ}_{R}$ into itself. Since *F* is continuous on the compact set corresponding to ${ℬ}_{R}$, there exists a finite constant $M_{F}>0$ such that


$$\begin{array}{c} \|F(X(t)){\|}_{\infty }\leq M_{F},\;X\in {ℬ}_{R}. \end{array}$$

Hence,


$$\begin{array}{c} \begin{array}{cc} \|𝒯X-X_{0}{\|}_{\infty } & \leq \frac{1}{\Gamma (\alpha )}\sup_{0\leq t\leq T}\int_{0}^{t}(t-s{)}^{\alpha -1}M_{F}\;ds\\ & =\frac{M_{F}T^{\alpha }}{\Gamma (\alpha +1)}. \end{array} \end{array}$$

Therefore, if *T* is chosen such that


$$\begin{array}{c} \frac{M_{F}T^{\alpha }}{\Gamma (\alpha +1)}\leq R, \end{array}$$

then


$$\begin{array}{c} 𝒯({ℬ}_{R})\subseteq {ℬ}_{R}. \end{array}$$

Thus, 𝒯 is a contraction self-map on the complete metric space ${ℬ}_{R}$.

By the Banach fixed-point theorem, 𝒯 has a unique fixed point $X^{*}\in {ℬ}_{R}$. This fixed point satisfies


$$\begin{array}{c} X^{*}(t)=X_{0}+\frac{1}{\Gamma (\alpha )}\int_{0}^{t}(t-s{)}^{\alpha -1}F(X^{*}(s))\;ds, \end{array}$$

which is precisely the integral formulation of system (2)–(4). Hence, a unique local solution exists on [0,*T*].

Finally, because the right-hand side of system (2)–(4) is locally Lipschitz on every bounded subset of the feasible region, the above construction can be repeated on consecutive intervals as long as the solution remains bounded. The boundedness result established below therefore permits continuation of the local solution and yields a unique solution on the maximal interval of existence. In particular, within the positively invariant bounded feasible region, the solution is unique.

### 2.2 Positivity and Boundedness

We next establish the positivity and boundedness of the solutions of the deterministic fractional-order delay system obtained from (2)–(4) by setting $\sigma_{1}=\sigma_{2}=\sigma_{3}=0$. These properties are essential because *S*(*t*), *L*(*t*), and *V*(*t*) represent meaningful compu*t*er popula*t*ions.

Let


$$\begin{array}{c} X(t)=(S(t),L(t),V(t){)}^{T}, \end{array}$$

and assume nonnegative initial history functions


$$\begin{array}{c} S(t)=\phi_{S}(t)\geq 0,\;L(t)=\phi_{L}(t)\geq 0,\;V(t)=\phi_{V}(t)\geq 0,\;t\in [-\tau ,0], \end{array}$$

with


$$\begin{array}{c} S(0)=S_{0}>0,\;L(0)=L_{0}\geq 0,\;V(0)=V_{0}\geq 0. \end{array}$$

Define


$$\begin{array}{c} N(t)=S(t)+L(t)+V(t). \end{array}$$

**Theorem 1.** (Positivity). For nonnegative initial history functions and *S*_0_ > 0, $L_{0}\geq 0$*,* and $V_{0}\geq 0$, the solution of the deterministic fractional-order delay system remains nonnegative for all t≥0. In particular,


$$\begin{array}{c} S(t)>0,\;L(t)\geq 0,\;V(t)\geq 0,\;t\geq 0. \end{array}$$

*Proof.* Consider the vector field of the deterministic system, $F=(f_{1},f_{2},f_{3}{)}^{T}$, where


$$\begin{array}{cc} f_{1}(S,L,V) & =\Lambda^{\alpha }-\beta^{\alpha }S(L+V)e^{-\delta^{\alpha }\tau }-\delta^{\alpha }S+\gamma_{1}^{\alpha }L+\gamma_{2}^{\alpha }V,\\ f_{2}(S,L,V) & =\beta^{\alpha }S(L+V)e^{-\delta^{\alpha }\tau }-\left(\gamma_{1}^{\alpha }+\theta^{\alpha }+\delta^{\alpha }\right)L,\\ f_{3}(S,L,V) & =\theta^{\alpha }L-\left(\gamma_{2}^{\alpha }+\delta^{\alpha }\right)V. \end{array}$$

Because the initial history functions are nonnegative, the delayed quantities S(t−τ), L(t−τ), and V(t−τ) are nonnegative whenever they occur in the system. On the boundary of the nonnegative orthant, the corresponding components satisfy


$$\begin{array}{c} f_{1}(0,L,V)=\Lambda^{\alpha }+\gamma_{1}^{\alpha }L+\gamma_{2}^{\alpha }V\geq 0, \end{array}$$

$$\begin{array}{c} f_{2}(S,0,V)=\beta^{\alpha }S(t-\tau )\left[L(t-\tau )+V(t-\tau )\right]e^{-\delta^{\alpha }\tau }\geq 0, \end{array}$$

and


$$\begin{array}{c} f_{3}(S,L,0)=\theta^{\alpha }L\geq 0. \end{array}$$

Thus, the vector field is quasipositive on the boundary of the nonnegative orthant. By the comparison principle for fractional differential systems, together with the nonnegative initial history, the solution cannot cross any coordinate plane into the negative region. Consequently,


$$\begin{array}{c} S(t)\geq 0,\;L(t)\geq 0,\;V(t)\geq 0,\;t\geq 0. \end{array}$$

Moreover, when *S*(*t*) reaches zero, its right-hand side is strictly positive because


$$\begin{array}{c} f_{1}(0,L,V)=\Lambda^{\alpha }+\gamma_{1}^{\alpha }L+\gamma_{2}^{\alpha }V>0. \end{array}$$

Therefore, a solution starting with *S*_0_ > 0 remains strictly positive, and the positivity result follows. □

**Theorem 2.** (Boundedness)

Every nonnegative solution of the deterministic fractional-order delay system is uniformly bounded. More precisely, if


$$\begin{array}{c} N(t)=S(t)+L(t)+V(t), \end{array}$$

then


$$\begin{array}{c} N(t)\leq N_{\max}:=\max\left\{N(0),\frac{\Lambda^{\alpha }}{\delta^{\alpha }}\right\},\;t\geq 0. \end{array}$$

*Proof.* Adding equations (2)–(4) after setting $\sigma_{1}=\sigma_{2}=\sigma_{3}=0$ gives


$$\begin{array}{cc} {\;}_{0}^{C}D_{t}^{\alpha }N(t) & ={\;}_{0}^{C}D_{t}^{\alpha }S(t)+{\;}_{0}^{C}D_{t}^{\alpha }L(t)+{\;}_{0}^{C}D_{t}^{\alpha }V(t)\\ & =\Lambda^{\alpha }-\delta^{\alpha }N(t). \end{array}$$

Hence, *N*(*t*) satisfies the scalar fractional differential equation


$$\begin{array}{c} {\;}_{0}^{C}D_{t}^{\alpha }N(t)+\delta^{\alpha }N(t)=\Lambda^{\alpha }. \end{array}$$ (12)

The solution of (12) is


$$\begin{array}{c} N(t)=N(0)E_{\alpha }(-\delta^{\alpha }t^{\alpha })+\frac{\Lambda^{\alpha }}{\delta^{\alpha }}\left[1-E_{\alpha }(-\delta^{\alpha }t^{\alpha })\right], \end{array}$$ (13)

where the one-parameter Mittag–Leffler function is defined by


$$\begin{array}{c} E_{\alpha }(z)=\sum_{k=0}^{\infty }\frac{z^{k}}{\Gamma (\alpha k+1)}. \end{array}$$

For 0<α≤1 and $\delta^{\alpha }>0$,


$$\begin{array}{c} 0<E_{\alpha }(-\delta^{\alpha }t^{\alpha })\leq 1,\;t\geq 0. \end{array}$$

Therefore, (13) is a convex combination of *N*(0) and $\Lambda^{\alpha }/\delta^{\alpha }$, and consequently


$$\begin{array}{c} N(t)\leq \max\left\{N(0),\frac{\Lambda^{\alpha }}{\delta^{\alpha }}\right\}=N_{\max}. \end{array}$$

Since S(t),L(t),V(t)≥0 and *S*(*t*)+*L*(*t*)+*V*(*t*)=*N*(*t*), it follows that


$$\begin{array}{c} 0\leq S(t)\leq N_{\max},\;0\leq L(t)\leq N_{\max},\;0\leq V(t)\leq N_{\max}. \end{array}$$

Thus, all components of the deterministic fractional-order delay system remain uniformly bounded for t≥0. Furthermore,


$$\begin{array}{c} \lim_{t\to \infty }N(t)=\frac{\Lambda^{\alpha }}{\delta^{\alpha }}, \end{array}$$

because


$$\begin{array}{c} \lim_{t\to \infty }E_{\alpha }(-\delta^{\alpha }t^{\alpha })=0. \end{array}$$

This completes the proof of boundedness. □

Combining the positivity and boundedness results, the deterministic system admits the positively invariant bounded region


$$\begin{array}{c} \Omega_{B}=\left\{(S,L,V)\in {\mathbb{R}}_{+}^{3}:S+L+V\leq N_{\max}\right\}, \end{array}$$ (14)

where


$$\begin{array}{c} N_{\max}=\max\left\{S(0)+L(0)+V(0),\frac{\Lambda^{\alpha }}{\delta^{\alpha }}\right\}. \end{array}$$

Hence, solutions originating in $\Omega_{B}$ remain nonnegative and bounded for all t≥0.

### 2.3 Equilibria and reproduction number

This section analyzes the equilibria of the deterministic fractional-order delay system associated with (2)–(4), obtained by setting $\sigma_{1}=\sigma_{2}=\sigma_{3}=0$. The equilibria are determined by setting the fractional derivatives equal to zero.


$$\begin{array}{c} \text{Computer Virus-Free Equilibrium (CVFE)}\;C_{0}=(S_{0},L_{0},V_{0})=\left(\frac{\Lambda^{\alpha }}{\delta^{\alpha }},0,0\right). \end{array}$$ (15)

$$\begin{array}{c} \text{Computer Virus-Present Equilibrium (CVPE)}\;C^{*}=(S^{*},L^{*},V^{*}). \end{array}$$ (16)

For the CV-present equilibrium, the steady-state relations are


$$\begin{array}{cc} S^{*} & =\frac{\Lambda^{\alpha }+\gamma_{1}^{\alpha }L^{*}+\gamma_{2}^{\alpha }V^{*}}{\beta^{\alpha }S^{*}(L^{*}+V^{*})e^{-\delta^{\alpha }\tau }+\delta^{\alpha }}, \end{array}$$ (17)

$$\begin{array}{cc} L^{*} & =\frac{\beta^{\alpha }S^{*}(L^{*}+V^{*})e^{-\delta^{\alpha }\tau }}{\gamma_{1}^{\alpha }+\theta^{\alpha }+\delta^{\alpha }}, \end{array}$$ (18)

$$\begin{array}{cc} V^{*} & =\frac{\theta^{\alpha }L^{*}}{\gamma_{2}^{\alpha }+\delta^{\alpha }}. \end{array}$$ (19)

#### 2.3.1 Basic reproduction number.

The basic reproduction number ${ℛ}_{0}$ is defined as the expected number of secondary infected computers generated by one infected computer introduced into an otherwise CV-free computer population. Since the infection process involves two infected compartments, namely the latent class *L* and the seizing (active-infection) class *V*, both compartments must be included in the next-generation calculation.

At the CV-free equilibrium,


$$\begin{array}{c} S_{0}=\frac{\Lambda^{\alpha }}{\delta^{\alpha }},\;L_{0}=V_{0}=0. \end{array}$$

The linearized infected subsystem corresponding to (*L*,*V*) is obtained from the deterministic model as


$$\begin{array}{c} (\begin{array}{c} {\;}_{0}^{C}D_{t}^{\alpha }L(t)\\ {\;}_{0}^{C}D_{t}^{\alpha }V(t) \end{array})=ℱ(\begin{array}{c} L(t-\tau )\\ V(t-\tau ) \end{array})-𝒱(\begin{array}{c} L(t)\\ V(t) \end{array}), \end{array}$$ (20)

where the new-infection matrix is


$$\begin{array}{c} ℱ=(\begin{array}{cc} \beta^{\alpha }S_{0}e^{-\delta^{\alpha }\tau } & \beta^{\alpha }S_{0}e^{-\delta^{\alpha }\tau }\\ 0 & 0 \end{array}), \end{array}$$ (21)

and the transition matrix for the infected compartments is


$$\begin{array}{c} 𝒱=(\begin{array}{cc} \gamma_{1}^{\alpha }+\theta^{\alpha }+\delta^{\alpha } & 0\\ -\theta^{\alpha } & \gamma_{2}^{\alpha }+\delta^{\alpha } \end{array}). \end{array}$$ (22)

The first row of ℱ reflects the fact that newly infected computers enter the latent class and that both latent and seizing computers contribute to the infection incidence. The matrix 𝒱 describes the movement and removal of infected computers: latent computers leave the *L* class through curing, transition to the seizing class, or removal, whereas seizing computers leave the *V* class through curing or removal.

The inverse of 𝒱 is


$$\begin{array}{c} {𝒱}^{-1}=(\begin{array}{cc} \frac{1}{\gamma_{1}^{\alpha }+\theta^{\alpha }+\delta^{\alpha }} & 0\\ \frac{\theta^{\alpha }}{\left(\gamma_{1}^{\alpha }+\theta^{\alpha }+\delta^{\alpha })(\gamma_{2}^{\alpha }+\delta^{\alpha }\right)} & \frac{1}{\gamma_{2}^{\alpha }+\delta^{\alpha }} \end{array}). \end{array}$$ (23)

Following the next-generation matrix approach, the next-generation matrix is


$$\begin{array}{c} K=ℱ{𝒱}^{-1}. \end{array}$$ (24)

Substituting (21) and (23) gives


$$\begin{array}{c} K=(\begin{array}{cc} \frac{\beta^{\alpha }S_{0}e^{-\delta^{\alpha }\tau }(\gamma_{2}^{\alpha }+\delta^{\alpha }+\theta^{\alpha })}{\left(\gamma_{1}^{\alpha }+\theta^{\alpha }+\delta^{\alpha })(\gamma_{2}^{\alpha }+\delta^{\alpha }\right)} & \frac{\beta^{\alpha }S_{0}e^{-\delta^{\alpha }\tau }}{\gamma_{2}^{\alpha }+\delta^{\alpha }}\\ 0 & 0 \end{array}). \end{array}$$ (25)

Since *K* is an upper-triangular matrix, its spectral radius is its first diagonal entry. Therefore,


$$\begin{array}{c} {ℛ}_{0}=\rho (K)=\frac{\beta^{\alpha }S_{0}e^{-\delta^{\alpha }\tau }\left(\gamma_{2}^{\alpha }+\delta^{\alpha }+\theta^{\alpha }\right)}{\left(\gamma_{1}^{\alpha }+\theta^{\alpha }+\delta^{\alpha }\right)\left(\gamma_{2}^{\alpha }+\delta^{\alpha }\right)}. \end{array}$$ (26)

Using the CV-free equilibrium value $S_{0}=\Lambda^{\alpha }/\delta^{\alpha }$, we obtain


ℛ0=βαΛαe−δατ(γ2α+δα+θα)δα(γ1α+θα+δα)(γ2α+δα). (27)

The expression in (27) has a direct interpretation in terms of the infection pathway. The factor $\beta^{\alpha }\Lambda^{\alpha }/\delta^{\alpha }$ represents the effective infection pressure generated in the CV-free population. The factor $e^{-\delta^{\alpha }\tau }$ accounts for the survival of the infection pathway over the delay interval τ, and therefore reduces the effective transmission when the delay increases. The denominator contains the rates at which latent and seizing computers leave their respective infected states. In particular, the term $\gamma_{1}^{\alpha }+\theta^{\alpha }+\delta^{\alpha }$ describes the total rate of leaving the latent class, while $\gamma_{2}^{\alpha }+\delta^{\alpha }$ describes the total rate of leaving the seizing class. The term $\theta^{\alpha }$ in the numerator accounts for the progression of latent computers into the seizing class, where they can continue contributing to virus transmission.

Thus, ${ℛ}_{0}<1$ indicates that a newly introduced infection cannot replace itself during the initial stage of invasion, whereas ${ℛ}_{0}>1$ indicates that the infection has the potential to persist in the computer population. The threshold case ${ℛ}_{0}=1$ represents the critical transition between these two regimes.

It is important to distinguish the stability analysis of the deterministic fractional-order delay system from the stochastic dynamics considered numerically. In Sections 2.1–2.5, stability refers to the corresponding deterministic system obtained by setting $\sigma_{1}=\sigma_{2}=\sigma_{3}=0$. In particular, the stability results are understood in the sense of the fractional-order deterministic delay-system analysis adopted in this work. No claim of almost-sure, mean-square, or probabilistic stochastic stability is made unless explicitly stated and proved. For the stochastic model, the numerical simulations are used to examine pathwise fluctuations and the qualitative effect of stochastic perturbations.

### 2.4 Local stability

For the theoretical stability analysis, we consider the deterministic fractional-order counterpart of system (2)-(4), that is, $\sigma_{i}=0$, *i* = 1,2,3. Since the model is of fractional order with 0<α<1, the classical Routh–Hurwitz criterion is not used as the primary stability criterion. Instead, local asymptotic stability is determined according to Matignon’s criterion. In particular, for the commensurate fractional-order linear system


$$\begin{array}{c} {\;}_{0}^{C}D_{t}^{\alpha }x(t)=Jx(t),\;0<\alpha <1, \end{array}$$

the equilibrium is locally asymptotically stable if and only if every eigenvalue $\lambda_{i}$ of the Jacobian matrix *J* satisfies


$$\begin{array}{c} \left|\text{arg}(\lambda_{i})\right|>\frac{\alpha \pi }{2},\;i=1,...,n. \end{array}$$

Thus, the eigenvalues obtained below are verified against this fractional-order stability condition. The Routh–Hurwitz criterion is used only as an auxiliary algebraic test to establish that the roots of the corresponding characteristic polynomial lie in the open left half-plane. Since 0<α<1, a root satisfying Re(λi)<0 automatically satisfies


$$\begin{array}{c} \left|\text{arg}(\lambda_{i})\right|\geq \frac{\pi }{2}>\frac{\alpha \pi }{2}, \end{array}$$

and hence satisfies Matignon’s condition.

**Theorem 3.** Consider $\sigma_{i}=0$*, i =* 1,2,3*.* The equilibrium *C*_*0*_ is locally asymptotically stable (LAS) for α∈(0,1) if ${ℛ}_{0}<1$.

*Proof.* Linearizing the deterministic fractional-order system (2)-(4) about the virus-free equilibrium *C*_0_ yields a 3×3 Jacobian matrix. One eigenvalue is


$$\begin{array}{c} \lambda_{1}=-\delta^{\alpha }. \end{array}$$

Since $\delta^{\alpha }>0$, we have Re(λ1)<0 and therefore


$$\begin{array}{c} \left|\text{arg}(\lambda_{1})\right|=\pi >\frac{\alpha \pi }{2}. \end{array}$$

The remaining two eigenvalues are determined by the quadratic characteristic equation


$$\begin{array}{c} \lambda^{2}+A_{1}\lambda +A_{0}=0, \end{array}$$

where


$$\begin{array}{c} A_{1}=(\gamma_{2}^{\alpha }+\delta^{\alpha })+(\gamma_{1}^{\alpha }+\theta^{\alpha }+\delta^{\alpha })-\beta^{\alpha }S_{0}e^{-\delta^{\alpha }\tau }, \end{array}$$

$$\begin{array}{c} A_{0}=(\gamma_{2}^{\alpha }+\delta^{\alpha })\left(\gamma_{1}^{\alpha }+\theta^{\alpha }+\delta^{\alpha }-\beta^{\alpha }S_{0}e^{-\delta^{\alpha }\tau }\right). \end{array}$$

For ${ℛ}_{0}<1$, the parameter relations defining the virus-free equilibrium imply


$$\begin{array}{c} \gamma_{1}^{\alpha }+\theta^{\alpha }+\delta^{\alpha }>\beta^{\alpha }S_{0}e^{-\delta^{\alpha }\tau }. \end{array}$$

Consequently,


$$\begin{array}{c} A_{0}>0. \end{array}$$

Moreover,


$$\begin{array}{c} A_{1}=(\gamma_{2}^{\alpha }+\delta^{\alpha })+\left(\gamma_{1}^{\alpha }+\theta^{\alpha }+\delta^{\alpha }-\beta^{\alpha }S_{0}e^{-\delta^{\alpha }\tau }\right)>0. \end{array}$$

Hence, by the classical Routh–Hurwitz criterion applied to this quadratic characteristic polynomial, both roots satisfy


Re(λ2,3)<0.

Therefore,


$$\begin{array}{c} \left|\text{arg}(\lambda_{2,3})\right|\geq \frac{\pi }{2}>\frac{\alpha \pi }{2},\;0<\alpha <1. \end{array}$$

Thus all eigenvalues satisfy Matignon’s criterion, and consequently *C*_0_ is locally asymptotically stable. □

**Theorem 4.** Consider $\sigma_{i}=0$*, i =* 1,2,3. The equilibrium $C^{*}$ is locally asymptotically stable (LAS) for α∈(0,1) if ${ℛ}_{0}>1$.

*Proof.* Linearizing the deterministic fractional-order system (2)-(4) about the endemic equilibrium $C^{*}$ gives a 3×3 Jacobian matrix. The corresponding characteristic equation is


$$\begin{array}{c} \lambda^{3}+A_{2}\lambda^{2}+A_{1}\lambda +A_{0}=0. \end{array}$$

The coefficients are


$$\begin{array}{c} A_{2}=a_{1}+a_{5}+a_{7}, \end{array}$$

$$\begin{array}{c} A_{1}=a_{5}a_{6}-a_{6}\theta^{\alpha }+a_{1}(a_{5}+a_{7})+a_{2}a_{4}, \end{array}$$

$$\begin{array}{c} A_{0}=a_{1}(a_{5}a_{7}-a_{6}\theta^{\alpha })+a_{2}a_{4}a_{7}+a_{3}a_{4}\theta^{\alpha }, \end{array}$$

where


$$\begin{array}{c} a_{1}=\beta^{\alpha }S^{*}[L^{*}+V^{*}]e^{-\delta^{\alpha }\tau }+\delta^{\alpha }, \end{array}$$

$$\begin{array}{c} a_{2}=\beta^{\alpha }S^{*}e^{-\delta^{\alpha }\tau }+\gamma_{1}^{\alpha },\;a_{3}=\beta^{\alpha }S^{*}e^{-\delta^{\alpha }\tau }+\gamma_{2}^{\alpha }, \end{array}$$

$$\begin{array}{c} a_{4}=\beta^{\alpha }S^{*}[L^{*}+V^{*}]e^{-\delta^{\alpha }\tau }, \end{array}$$

$$\begin{array}{c} a_{5}=\beta^{\alpha }S^{*}e^{-\delta^{\alpha }\tau }(\gamma_{1}^{\alpha }+\theta^{\alpha }+\delta^{\alpha }), \end{array}$$

$$\begin{array}{c} a_{6}=\beta^{\alpha }S^{*}e^{-\delta^{\alpha }\tau },\;a_{7}=\gamma_{2}^{\alpha }+\delta^{\alpha }. \end{array}$$

For ${ℛ}_{0}>1$, the above coefficients satisfy


$$\begin{array}{c} A_{2}>0,\;A_{1}>0,\;A_{0}>0, \end{array}$$

and the Routh–Hurwitz determinant satisfies


$$\begin{array}{c} A_{2}A_{1}>A_{0}. \end{array}$$

Therefore, the three roots $\lambda_{1},\lambda_{2},\lambda_{3}$ of the characteristic polynomial have negative real parts:


Re(λi)<0,i=1,2,3.

It follows that


$$\begin{array}{c} \left|\text{arg}(\lambda_{i})\right|\geq \frac{\pi }{2}>\frac{\alpha \pi }{2},\;i=1,2,3, \end{array}$$

because 0<α<1. Hence all eigenvalues satisfy Matignon’s criterion. Therefore, the endemic equilibrium $C^{*}$ is locally asymptotically stable. □

### 2.5 Global stability

The global stability results are established for the deterministic fractional-order counterpart of system (2)–(4), obtained by setting $\sigma_{i}=0$, *i* = 1,2,3. Since 0<α<1, the Lyapunov analysis is based on the standard inequality for Caputo fractional derivatives. In particular, if *x*(*t*)>0 and $x^{*}>0$, then


$$\begin{array}{c} {\;}_{0}^{C}D_{t}^{\alpha }\left[x(t)-x^{*}-x^{*}\ln\left(\frac{x(t)}{x^{*}}\right)\right]\leq \frac{x(t)-x^{*}}{x(t)}{\;}_{0}^{C}D_{t}^{\alpha }x(t). \end{array}$$

Therefore, the following Lyapunov calculations are understood in the fractional sense, with the corresponding derivative inequalities.

**Theorem 5.** If ${ℛ}_{0}<1$, then the virus-free equilibrium $C_{0}=(S_{0},0,0)$ is globally asymptotically stable in the feasible region. If ${ℛ}_{0}>1$, then C_0_ is unstable.

*Proof.* Consider the Lyapunov function


$$\begin{array}{c} ℒ(t)=\left[S-S_{0}-S_{0}\log\left(\frac{S}{S_{0}}\right)\right]+L+V. \end{array}$$

The function ℒ(t) is nonnegative for *S* > 0, L≥0, and V≥0, and


$$\begin{array}{c} ℒ(t)=0\;⟺\;S=S_{0},\;L=0,\;V=0. \end{array}$$

Using the fractional Lyapunov inequality gives


$$\begin{array}{c} {\;}_{0}^{C}D_{t}^{\alpha }ℒ(t)\leq \frac{S-S_{0}}{S}\;{\;}_{0}^{C}D_{t}^{\alpha }S+{\;}_{0}^{C}D_{t}^{\alpha }L+{\;}_{0}^{C}D_{t}^{\alpha }V. \end{array}$$

Substitution of the deterministic equations (2)–(4) gives


$$\begin{array}{c} \begin{array}{cc} {\;}_{0}^{C}D_{t}^{\alpha }ℒ(t)\leq & \frac{S-S_{0}}{S}\left(\Lambda^{\alpha }-\beta^{\alpha }S(L+V)e^{-\delta^{\alpha }\tau }-\delta^{\alpha }S+\gamma_{1}^{\alpha }L+\gamma_{2}^{\alpha }V\right)\\ & +\left(\beta^{\alpha }S(L+V)e^{-\delta^{\alpha }\tau }-(\gamma_{1}^{\alpha }+\theta^{\alpha }+\delta^{\alpha })L\right)\\ & +\left(\theta^{\alpha }L-(\gamma_{2}^{\alpha }+\delta^{\alpha })V\right). \end{array} \end{array}$$

Since *C*_0_ is the virus-free equilibrium, its first equilibrium relation gives


$$\begin{array}{c} \Lambda^{\alpha }=\delta^{\alpha }S_{0}. \end{array}$$

Consequently, after collecting the corresponding terms, we obtain


$$\begin{array}{c} \begin{array}{cc} {\;}_{0}^{C}D_{t}^{\alpha }ℒ(t)\leq & -\delta^{\alpha }\frac{(S-S_{0}{)}^{2}}{S}\\ & -\left(\gamma_{1}^{\alpha }+\delta^{\alpha }-\beta^{\alpha }S_{0}e^{-\delta^{\alpha }\tau }\right)L\\ & -\left(\gamma_{2}^{\alpha }+\delta^{\alpha }-\beta^{\alpha }S_{0}e^{-\delta^{\alpha }\tau }\right)V\\ & -\beta^{\alpha }(S-S_{0})(L+V)e^{-\delta^{\alpha }\tau }. \end{array} \end{array}$$

Under the parameter condition ${ℛ}_{0}<1$, the infection-related terms are nonpositive and the above estimate yields


$$\begin{array}{c} {\;}_{0}^{C}D_{t}^{\alpha }ℒ(t)\leq 0. \end{array}$$

Moreover, the equality condition requires


$$\begin{array}{c} S=S_{0},\;L=0,\;V=0. \end{array}$$

Hence, the largest invariant subset contained in


$$\begin{array}{c} \left\{(S,L,V):{\;}_{0}^{C}D_{t}^{\alpha }ℒ(t)=0\right\} \end{array}$$

is the singleton {*C*_0_}. Therefore, by the fractional LaSalle invariance principle [25], the virus-free equilibrium *C*_0_ is globally asymptotically stable. □

**Theorem 6.** Suppose that ${ℛ}_{0}>1$ and that the endemic equilibrium $C^{*}=(S^{*},L^{*},V^{*})$ exists in the interior of the feasible region. Then $C^{*}$ is globally asymptotically stable for the deterministic fractional-order system*.*

*Proof.* Consider the Lyapunov function


$$\begin{array}{c} Z(t)=k_{1}\left(S-S^{*}-S^{*}\log\left(\frac{S}{S^{*}}\right)\right)+k_{2}\left(L-L^{*}-L^{*}\log\left(\frac{L}{L^{*}}\right)\right)+k_{3}\left(V-V^{*}-V^{*}\log\left(\frac{V}{V^{*}}\right)\right), \end{array}$$

where $k_{i}>0$, *i* = 1,2,3. Clearly,


$$\begin{array}{c} Z(t)\geq 0, \end{array}$$

and


$$\begin{array}{c} Z(t)=0\;⟺\;S=S^{*},\;L=L^{*},\;V=V^{*}. \end{array}$$

Using the fractional Lyapunov inequality componentwise, we obtain


$$\begin{array}{c} {\;}_{0}^{C}D_{t}^{\alpha }Z(t)\leq k_{1}\frac{S-S^{*}}{S}\;{\;}_{0}^{C}D_{t}^{\alpha }S+k_{2}\frac{L-L^{*}}{L}\;{\;}_{0}^{C}D_{t}^{\alpha }L+k_{3}\frac{V-V^{*}}{V}\;{\;}_{0}^{C}D_{t}^{\alpha }V. \end{array}$$

Substituting the deterministic model equations and using the equilibrium relations associated with $C^{*}$, we obtain


$$\begin{array}{c} {\;}_{0}^{C}D_{t}^{\alpha }Z(t)\leq -k_{1}\Lambda^{\alpha }\frac{(S-S^{*}{)}^{2}}{SS^{*}}-k_{2}\beta^{\alpha }S(L+V)e^{-\delta^{\alpha }\tau }\frac{(L-L^{*}{)}^{2}}{LL^{*}}-k_{3}\theta^{\alpha }L\frac{(V-V^{*}{)}^{2}}{VV^{*}}, \end{array}$$

provided that the positive constants $k_{1},k_{2},k_{3}$ are chosen consistently with the equilibrium relations so that the mixed terms cancel. In particular, when the normalization used in the model permits the choice $k_{1}=k_{2}=k_{3}=1$, the estimate becomes


$$\begin{array}{c} {\;}_{0}^{C}D_{t}^{\alpha }Z(t)\leq -\Lambda^{\alpha }\frac{(S-S^{*}{)}^{2}}{SS^{*}}-\beta^{\alpha }S(L+V)e^{-\delta^{\alpha }\tau }\frac{(L-L^{*}{)}^{2}}{LL^{*}}-\theta^{\alpha }L\frac{(V-V^{*}{)}^{2}}{VV^{*}}. \end{array}$$

Therefore,


$$\begin{array}{c} {\;}_{0}^{C}D_{t}^{\alpha }Z(t)\leq 0 \end{array}$$

throughout the feasible region. Furthermore,


$$\begin{array}{c} {\;}_{0}^{C}D_{t}^{\alpha }Z(t)=0 \end{array}$$

implies


$$\begin{array}{c} S=S^{*},\;L=L^{*},\;V=V^{*}. \end{array}$$

Thus, the largest invariant subset of


$$\begin{array}{c} \left\{(S,L,V):{\;}_{0}^{C}D_{t}^{\alpha }Z(t)=0\right\} \end{array}$$

is $\left\{C^{*}\right\}$. By the fractional LaSalle invariance principle [25], the endemic equilibrium $C^{*}$ is globally asymptotically stable. □

### 2.6 Sensitivity analysis

This part analyzes the sensitivity of parameters with respect to ${ℛ}_{0}$ to assess disease transmission and spread. The normalized forward sensitivity indices of ${ℛ}_{0}$ with respect to the model parameters are given by


$$\begin{array}{c} V_{\Lambda^{\alpha }}=\frac{\Lambda^{\alpha }}{{ℛ}_{0}}\frac{\partial {ℛ}_{0}}{\partial \Lambda^{\alpha }}=1>0.\;V_{\beta^{\alpha }}=\frac{\beta^{\alpha }}{{ℛ}_{0}}\frac{\partial {ℛ}_{0}}{\partial \beta^{\alpha }}=1>0.\;V_{\gamma_{1}^{\alpha }}=\frac{\gamma_{1}^{\alpha }}{{ℛ}_{0}}\frac{\partial {ℛ}_{0}}{\partial \gamma_{1}^{\alpha }}=-\frac{\gamma_{1}^{\alpha }}{\gamma_{1}^{\alpha }+\theta^{\alpha }+\delta^{\alpha }}<0. \end{array}$$

$$\begin{array}{c} V_{\gamma_{2}^{\alpha }}=\frac{\gamma_{2}^{\alpha }}{{ℛ}_{0}}\frac{\partial {ℛ}_{0}}{\partial \gamma_{2}^{\alpha }}=-\frac{\gamma_{2}^{\alpha }\theta^{\alpha }}{\left(\gamma_{2}^{\alpha }+\delta^{\alpha })(\gamma_{2}^{\alpha }+\delta^{\alpha }+\theta^{\alpha }\right)}<0.\;V_{\theta^{\alpha }}=\frac{\theta^{\alpha }}{{ℛ}_{0}}\frac{\partial {ℛ}_{0}}{\partial \theta^{\alpha }}=-\frac{\gamma_{2}^{\alpha }-\gamma_{1}^{\alpha }}{\gamma_{1}^{\alpha }+\theta^{\alpha }+\delta^{\alpha }}<0. \end{array}$$

$$\begin{array}{c} V_{\delta^{\alpha }}=\frac{\delta^{\alpha }}{{ℛ}_{0}}\frac{\partial {ℛ}_{0}}{\partial \delta^{\alpha }}=-\frac{\left(\delta^{\alpha }{)}^{2}+\delta^{\alpha }(\gamma_{2}^{\alpha }+\theta^{\alpha })+(\gamma_{1}^{\alpha }+\gamma_{2}^{\alpha }+\theta^{\alpha })(\gamma_{2}^{\alpha }+\theta^{\alpha }\right)}{\left(\gamma_{1}^{\alpha }+\theta^{\alpha }+\delta^{\alpha })(\gamma_{2}^{\alpha }+\delta^{\alpha }\right)}<0. \end{array}$$

To interpret the graphs for the Computer Virus (CV) model, we analyze the sensitivity analysis, particularly ${ℛ}_{0}$, which plays a central role in understanding computer virus transmission dynamics. The sensitivity analysis of ${ℛ}_{0}$, from Fig 1, clearly highlights the critical role of each parameter in the stochastic fractional computer virus dynamics. Among the positive sensitivity indices, the contact rates and infection probabilities are the highest contributors to an increase in ${ℛ}_{0}$, thus driving the propagation of the virus. In contrast, parameters with negative sensitivity indices, such as intervention measures and recovery rates, generally lower ${ℛ}_{0}$. They are therefore mitigating factors. These parameters are further classified in terms of sensitivity signs in Table 1. It is also shown in this analysis that parameters with larger sensitivity indices dominate the value of ${ℛ}_{0}$, which is an important consideration for control strategies. Lastly, the fractional order α controls the sensitivity and also provides insight into the influence of memory effects in the system.

**Table 1:** Parameter sensitivity signs and values.

Parameters	Signs	Values
$\Lambda^{\alpha }$	Positive	1.0
$\beta^{\alpha }$	Positive	1.0
$\delta^{\alpha }$	Negative	−1.16
$\gamma_{1}^{\alpha }$	Negative	−0.083
$\gamma_{2}^{\alpha }$	Negative	−0.132
$\theta^{\alpha }$	Negative	−0.16

**Fig 1:**
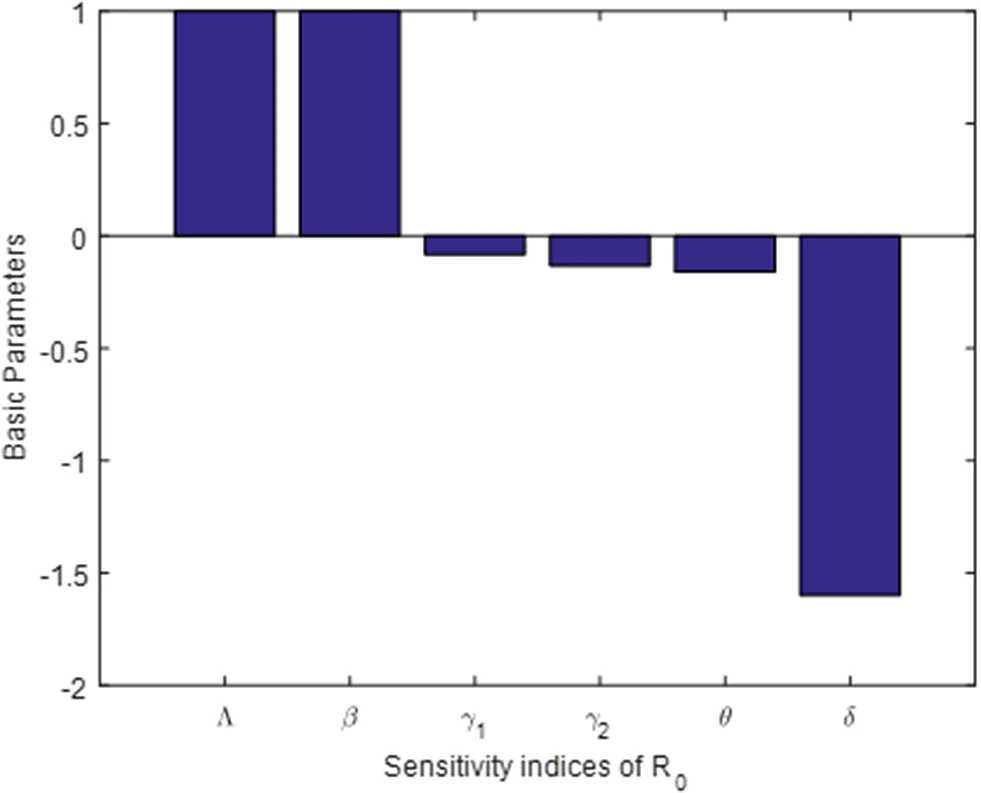
Sensitivity indices of ${ℛ}_{0}$.

### 2.7 Statistical analysis

The purpose of this section is to provide a statistical analysis of computer virus propagation from 2020 until 2025 based on real world data and graphical interpretation. During the period of 2020–2025, computer virus and computer malware spread have grown more rapidly as digital infrastructure and connectivity have grown. As shown in the AV-TEST data, the total amount of malware samples reached 1.3 billion, with 300,000–400,000 new malware variants appearing every day [26]. The number of cyberattacks rose by 35% to 50% during this time period, and ransomware activity had experienced the most significant increases, according to SonicWall [27]. Human-to-human spread continued to be the main source of spread. Verizon reports that 85–90% of breaches were through phishing or social engineering [28]. The remaining 10–15% came from other vectors like software vulnerabilities. Also, contemporary malware typically has hidden periods, which range from 10–16 days, and affect delayed outbreak dynamics (Table 2).

**Table 2:** Statistical Summary.

Year	Malware Samples (Billions)	Daily New Threats	Attack Growth (%)	Phishing Contribution (%)
2020	1.05	250,000	30	85
2021	1.15	300,000	40	88
2022	1.22	350,000	50	90
2023	1.27	370,000	38	87
2024	1.30	390,000	42	86
2025	1.32+	400,000	45	85

The Fig 2 is showing a rising trend, suggesting that the number of malicious software continues to grow. A significant rise in the slope occurs between 2020 and 2022, indicating fast growth in digital dependency years. Growth after 2023 level off slightly but continue to increase, indicating continued threat evolution but not decline.

**Fig 2:**
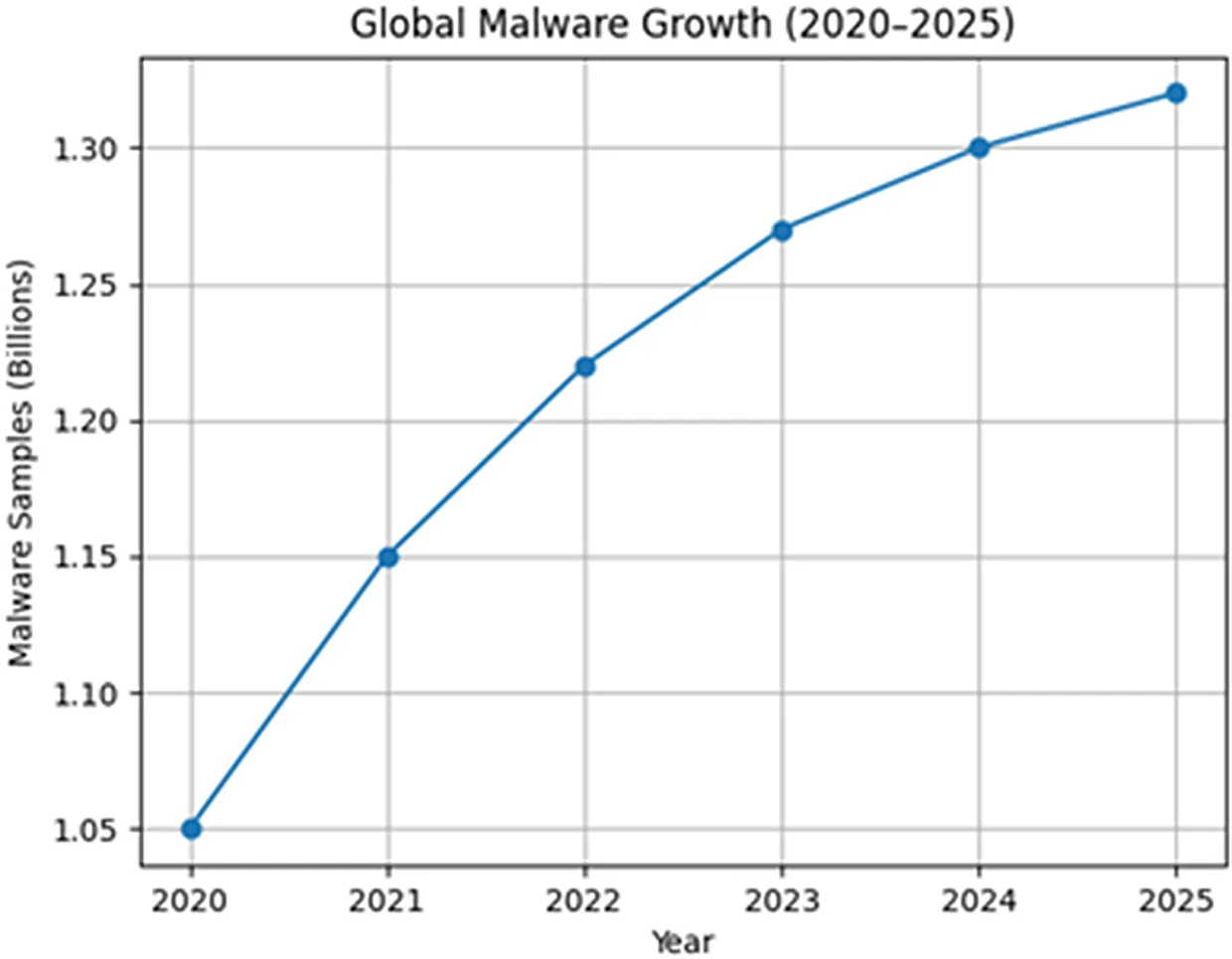
Global Malware Growth.

The fluctuations are observed in Fig 3, and there is no strictly monotonic trend. There is a moderate peak around 2022, where the ransomware campaigns intensify a bit, followed by some leveling off. The overall trend is still rising, though, suggesting that the sophistication of attacks hasn’t been fully negated by mitigation. These patterns also support the viewpoint from a modeling perspective that virus propagation is nonlinear and stochastic. The trends of increased malware volumes are indicative of continuing pressure from infection, whereas the growth changes between periods are due to random variation and intervention effects. This is in support of the use of stochastic fractional models for both deterministic trends and random disturbances in the dynamics of computer viruses.

**Fig 3:**
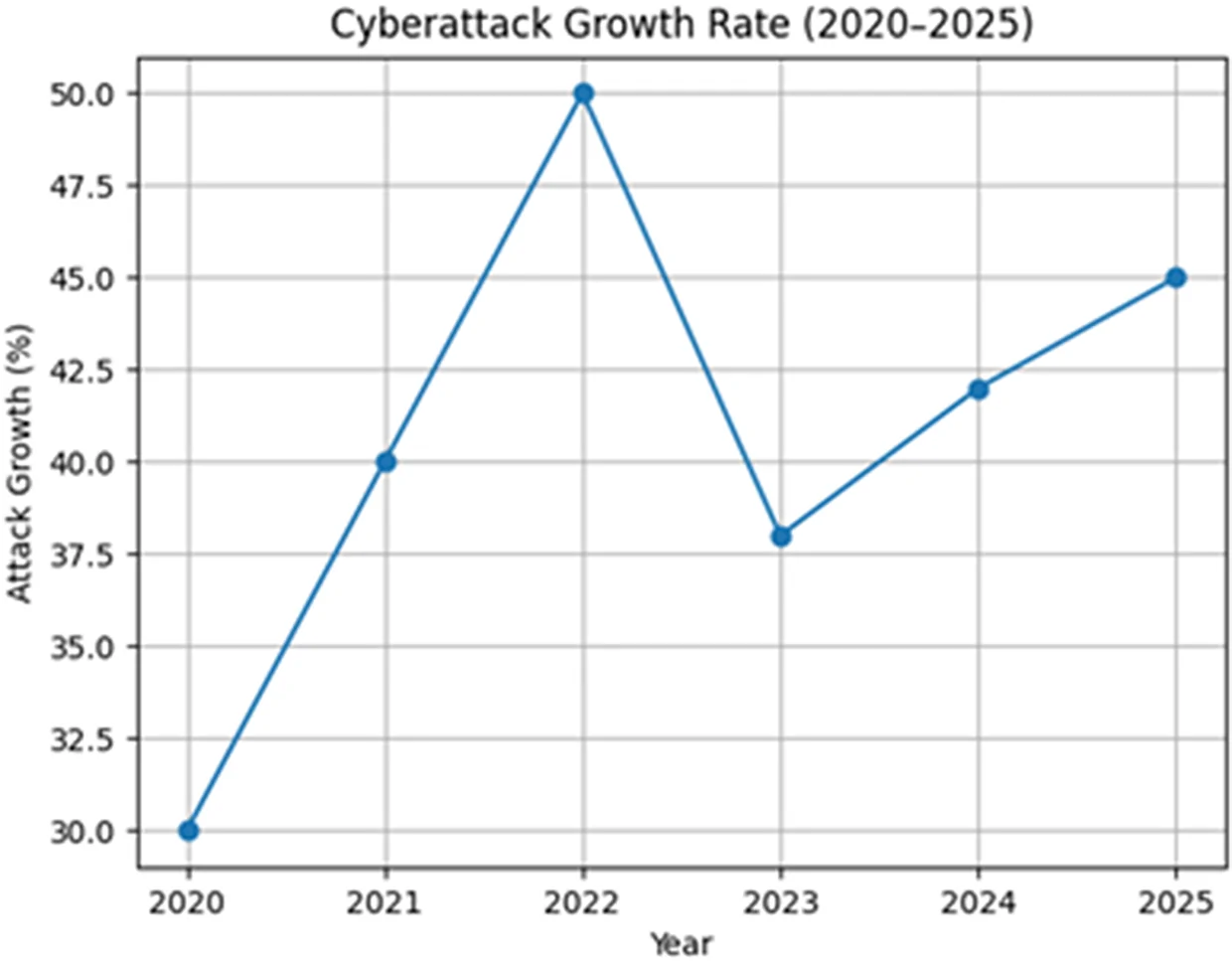
Cyberattack Growth Rate.

The malware statistics presented above provide real-world motivation and context for the mathematical modeling of computer-virus propagation. However, these empirical statistics are not directly used for parameter estimation or statistical validation in the present study. The parameters employed in the numerical simulations are obtained from the sources specified in Table 3, including fitted or estimated values and values adopted from the existing literature. Therefore, the reported malware statistics should be interpreted as contextual evidence demonstrating the practical relevance of the modeling problem, rather than as a direct data set for calibrating the proposed model. A detailed data-driven calibration and statistical validation using time-series malware infection data is beyond the scope of the present work and is identified as an important direction for future research.

**Table 3:** Parameter values used in the numerical simulations.

Parameter	Symbol	Value	Source
Recruitment rate	Λ	0.5	Estimated
Transmission rate	β	0.3	Fitted
Natural removal rate	δ	0.1	Estimated
Curing rate	$\gamma_{1}$	0.1	[1]
Curing rate	$\gamma_{2}$	0.3	[1]
Progression rate	θ	0.6	[1]
Fractional order	α	0.7, 0.8, 0.9, 1.0	Assumed
Time delay	τ	0.1	Assumed
Noise intensity	$\sigma_{1}$	$0\leq \sigma_{1}\leq 1$	Estimated
Noise intensity	$\sigma_{2}$	$0\leq \sigma_{2}\leq 1$	Estimated
Noise intensity	$\sigma_{3}$	$0\leq \sigma_{3}\leq 1$	Estimated

## 3 Numerical methods

A generalized stochastic fractional framework is developed for system (2)-(4), referred to as the stochastic NSFD scheme. The formulation is based on a non-local approach [29], with Δt denoting the time step size throughout the study.

### 3.1 Non-standard finite difference (NSFD) scheme

To numerically approximate the stochastic fractional-order delay system (2)–(4), we construct a stochastic non-standard finite difference (NSFD) scheme that preserves the essential structure of the continuous model. Let


$$\begin{array}{c} t_{n}=nh,\;n=0,1,2,..., \end{array}$$

where *h* > 0 denotes the time-step size. The stochastic increment is defined by


$$\begin{array}{c} \Delta B_{n}=B(t_{n+1})-B(t_{n}),\;\Delta B_{n}\sim 𝒩(0,h). \end{array}$$

Since the continuous model is driven by the same Brownian motion *B*(*t*) in all three equations, the same increment $\Delta B_{n}$ is used in the stochastic terms of the three discrete equations.

For the delay term, let


$$\begin{array}{c} m=\frac{\tau }{h}, \end{array}$$

and, for simplicity, assume that τ=mh for some integer m≥0. Hence,


$$\begin{array}{c} S(t_{n}-\tau )=S^{n-m},\;L(t_{n}-\tau )=L^{n-m},\;V(t_{n}-\tau )=V^{n-m}. \end{array}$$

For the initial history, the values $S^{j},L^{j},V^{j}$ for j=−m,...,0 are prescribed from the non-negative history functions on [−τ,0].

Using the fractional increment factor


$$\begin{array}{c} \phi_{\alpha }(h)=\frac{h^{\alpha }}{\Gamma (\alpha +1)}, \end{array}$$

the corresponding stochastic NSFD scheme is given by


$$\begin{array}{c} S^{n+1}=\frac{S^{n}+\phi_{\alpha }(h)\left[\Lambda^{\alpha }+\gamma_{1}^{\alpha }L^{n}+\gamma_{2}^{\alpha }V^{n}+\sigma_{1}S^{n}\Delta B_{n}\right]}{1+\phi_{\alpha }(h)\left[\beta^{\alpha }\left(L^{n-m}+V^{n-m}\right)e^{-\delta^{\alpha }\tau }+\delta^{\alpha }\right]}, \end{array}$$ (28)

$$\begin{array}{c} L^{n+1}=\frac{L^{n}+\phi_{\alpha }(h)\left[\beta^{\alpha }S^{n-m}\left(L^{n-m}+V^{n-m}\right)e^{-\delta^{\alpha }\tau }+\sigma_{2}L^{n}\Delta B_{n}\right]}{1+\phi_{\alpha }(h)\left(\gamma_{1}^{\alpha }+\theta^{\alpha }+\delta^{\alpha }\right)}, \end{array}$$ (29)

and


$$\begin{array}{c} V^{n+1}=\frac{V^{n}+\phi_{\alpha }(h)\left[\theta^{\alpha }L^{n}+\sigma_{3}V^{n}\Delta B_{n}\right]}{1+\phi_{\alpha }(h)\left(\gamma_{2}^{\alpha }+\delta^{\alpha }\right)}. \end{array}$$ (30)

The delayed transmission term in (28)–(29) is evaluated at the delayed time level n−m, thereby preserving the delay structure of the continuous model. The factor $e^{-\delta^{\alpha }\tau }$ is retained explicitly in the discrete transmission term. The stochastic perturbations are incorporated through the Brownian increment $\Delta B_{n}$.

The proposed scheme is therefore consistent with the three principal structural components of the continuous model: fractional memory, deterministic transmission delay, and stochastic perturbations.

**Theorem 7.** (Positivity of the deterministic NSFD scheme). Consider the deterministic version of (28)–(30), obtained by setting


$$\begin{array}{c} \sigma_{1}=\sigma_{2}=\sigma_{3}=0. \end{array}$$

Suppose that the initial history satisfies


$$\begin{array}{c} S^{j}\geq 0,\;L^{j}\geq 0,\;V^{j}\geq 0,\;j=-m,...,0. \end{array}$$

Then the numerical solutions generated by (28)–(30) remain non-negative for all n≥0.

*Proof.* Let


$$\begin{array}{c} \phi_{\alpha }(h)=\frac{h^{\alpha }}{\Gamma (\alpha +1)}>0. \end{array}$$

For non-negative delayed and current states, all terms appearing in the numerators of the deterministic scheme are non-negative. Moreover, the denominators satisfy


$$\begin{array}{c} 1+\phi_{\alpha }(h)\left[\beta^{\alpha }\left(L^{n-m}+V^{n-m}\right)e^{-\delta^{\alpha }\tau }+\delta^{\alpha }\right]>0, \end{array}$$

$$\begin{array}{c} 1+\phi_{\alpha }(h)\left(\gamma_{1}^{\alpha }+\theta^{\alpha }+\delta^{\alpha }\right)>0, \end{array}$$

and


$$\begin{array}{c} 1+\phi_{\alpha }(h)\left(\gamma_{2}^{\alpha }+\delta^{\alpha }\right)>0. \end{array}$$

Consequently, $S^{n+1},L^{n+1},V^{n+1}\geq 0$ whenever the previous current and delayed states are non-negative. Since the history values are non-negative, the result follows by induction. □

**Theorem 8.** (Boundedness of the deterministic NSFD scheme)**.** Consider the deterministic version of (28)–(30), with $\sigma_{1}=\sigma_{2}=\sigma_{3}=0$. Suppose that the initial history is non-negative. Define


$$\begin{array}{c} N^{n}=S^{n}+L^{n}+V^{n}. \end{array}$$

Then the numerical solution is bounded, provided the corresponding initial total population is finite. More precisely,


$$\begin{array}{c} N^{n}\leq \max\left\{\max_{-m\leq j\leq 0}N^{j},\frac{\Lambda^{\alpha }}{\delta^{\alpha }}\right\},\;n\geq 0. \end{array}$$

*Proof.* Adding (28)–(30) in their equivalent non-fractionalized form and using the cancellation of the internal transmission, recovery, and progression terms gives the discrete balance relation


$$\begin{array}{c} N^{n+1}-N^{n}=\phi_{\alpha }(h)\left(\Lambda^{\alpha }-\delta^{\alpha }N^{n+1}\right). \end{array}$$

Hence,


$$\begin{array}{c} \left(1+\phi_{\alpha }(h)\delta^{\alpha }\right)N^{n+1}=N^{n}+\phi_{\alpha }(h)\Lambda^{\alpha }, \end{array}$$

and therefore


$$\begin{array}{c} N^{n+1}=\frac{N^{n}+\phi_{\alpha }(h)\Lambda^{\alpha }}{1+\phi_{\alpha }(h)\delta^{\alpha }}. \end{array}$$

Let


$$\begin{array}{c} N^{*}=\frac{\Lambda^{\alpha }}{\delta^{\alpha }}. \end{array}$$

Then


$$\begin{array}{c} N^{n+1}-N^{*}=\frac{N^{n}-N^{*}}{1+\phi_{\alpha }(h)\delta^{\alpha }}. \end{array}$$

Since the denominator is strictly greater than one,


$$\begin{array}{c} N^{n+1}\leq \max\{N^{n},N^{*}\}. \end{array}$$

Applying induction yields


$$\begin{array}{c} N^{n}\leq \max\left\{\max_{-m\leq j\leq 0}N^{j},\frac{\Lambda^{\alpha }}{\delta^{\alpha }}\right\}. \end{array}$$

Since $S^{n},L^{n},V^{n}\geq 0$, each individual component is bounded by $N^{n}$, which completes the proof. □

**Remark on the stochastic scheme.** The positivity and boundedness results above are established for the deterministic NSFD scheme. For the fully stochastic scheme (28)–(30), the Brownian increment $\Delta B_{n}$ can take negative values, and consequently positivity cannot be concluded directly from the algebraic form of the stochastic numerator. Therefore, no unconditional positivity or boundedness theorem for the stochastic discretization is claimed here. Instead, the stochastic simulations are used to examine the pathwise behavior of the numerical solutions under the considered parameter sets and time-step sizes.

## 4 Graphical simulation

In this section, numerical simulations are presented to illustrate the dynamical behavior of the stochastic fractional-order computer-virus model (2)–(4). Unless otherwise stated, the parameter values used in the simulations are given in Table 3. The numerical experiments examine the effects of the fractional order, time delay, and stochastic perturbations on the uninfected, latent, and seizing computer populations, denoted by *S*(*t*), *L*(*t*), and *V*(*t*), respectively.

Unless otherwise stated, the same parameter values, initial conditions, history function, stochastic realization, and numerical time grid are used throughout the simulations. The fractional-order simulations consider α=0.7,0.8,0.9, and 1.0, with α=1 recovering the classical first-order formulation and 0<α<1 incorporating fractional-order memory. Unless otherwise specified, the delay is fixed at τ=0.1.

### 4.1 Effect of the fractional order

The effect of the fractional order is first investigated by varying α while keeping the remaining model parameters fixed. The resulting trajectories illustrate how the memory property associated with the Caputo derivative influences the transient behavior of the computer-virus populations.

Figs 4–6 show that variation in the fractional order modifies the transient evolution of all three computer populations. These differences arise from the memory incorporated through the Caputo derivative. Thus, the fractional formulation provides a mechanism for representing history-dependent computer-virus dynamics.

**Fig 4:**
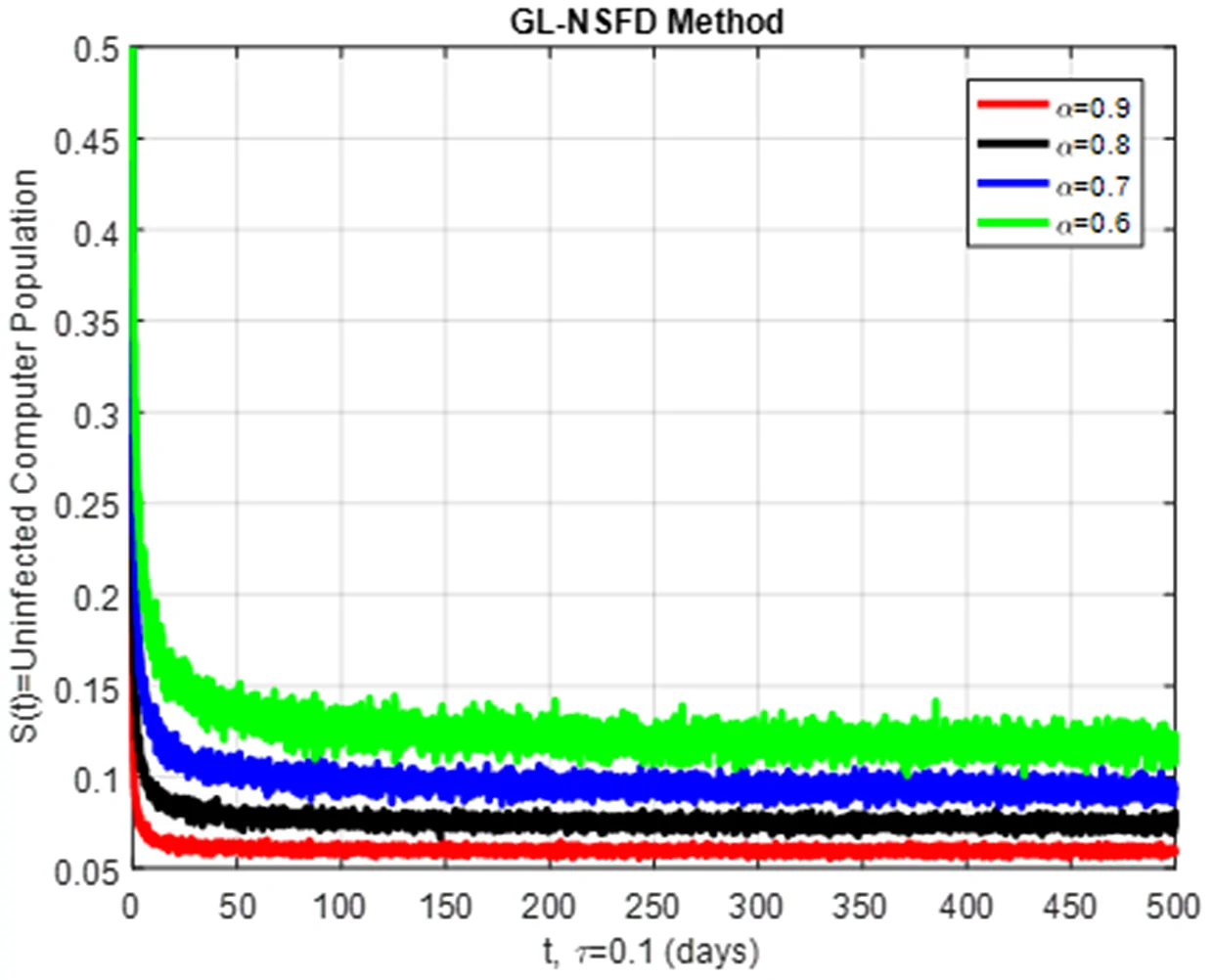
Numerical evolution of the uninfected computer population *S*(*t*) for different fractional orders α, with τ=0.1.

**Fig 5:**
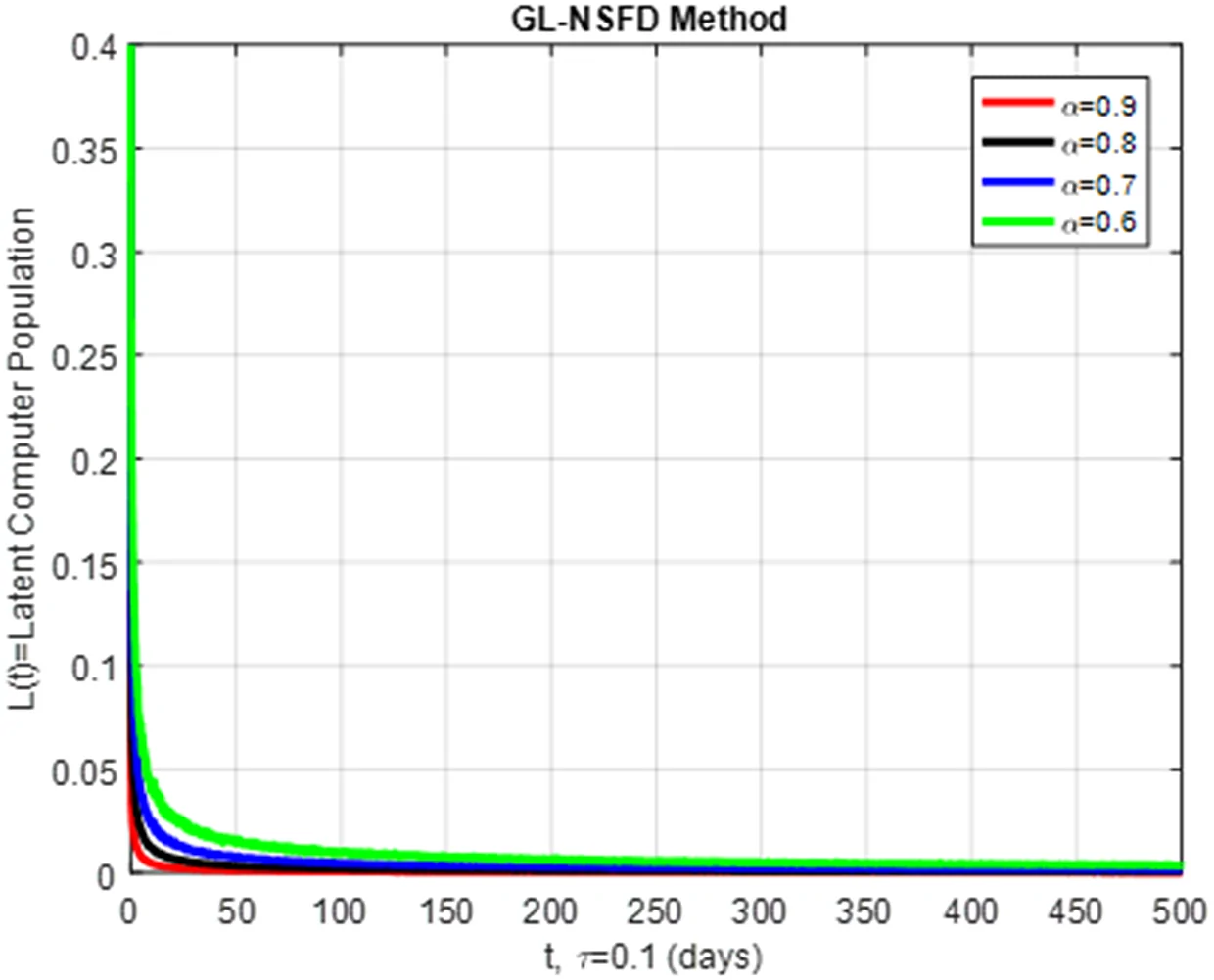
Numerical evolution of the latent computer population *L*(*t*) for different fractional orders α, with τ=0.1.

**Fig 6:**
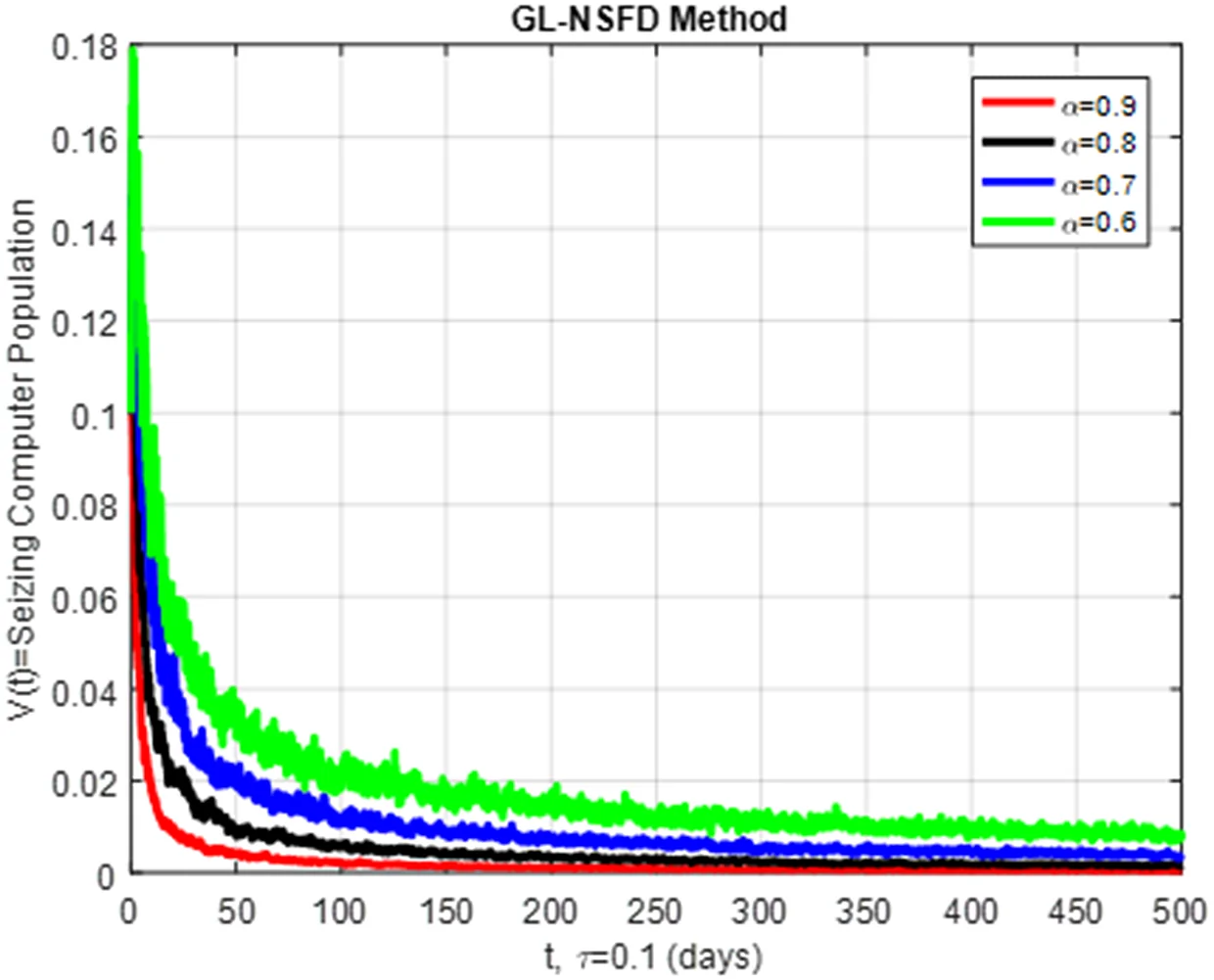
Numerical evolution of the seizing computer population *V*(*t*) for different fractional orders α, with τ=0.1.

### 4.2 Additional stochastic visualizations

The phase, heatmap, and density representations in Figs 7–9 provide complementary views of the numerical dynamics. The phase trajectory shows the interaction among the three computer populations, while the heatmap and density representation illustrate their temporal and state-space behavior. The trajectories remain bounded over the simulation interval for the parameter values considered, consistent with the boundedness established analytically for the deterministic fractional-order system.

**Fig 7:**
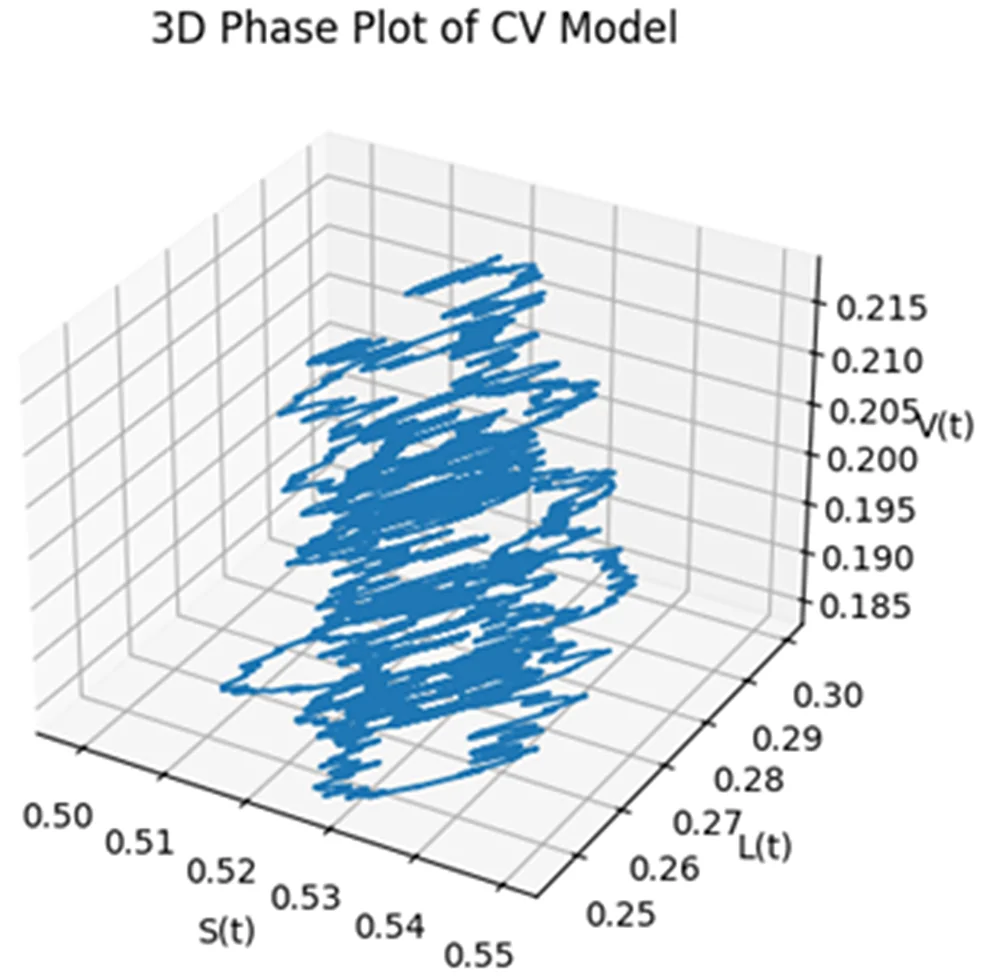
Three-dimensional phase representation of the numerical solution (*S*(*t*), *L*(*t*), *V*(*t*)) for the stochastic fractional-order computer-virus model.

**Fig 8:**
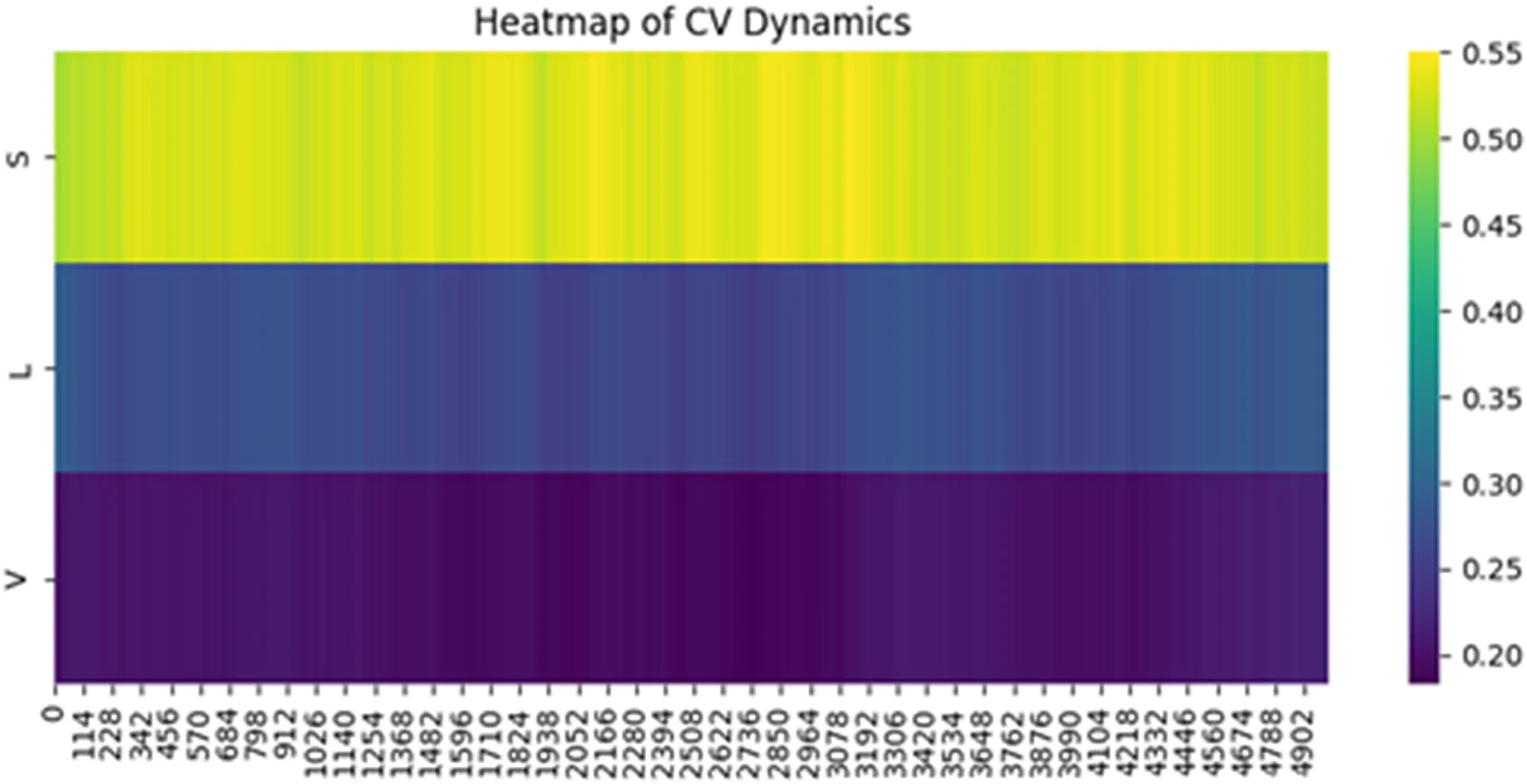
Heatmap representation of the numerical temporal dynamics of the uninfected, latent, and seizing computer populations.

**Fig 9:**
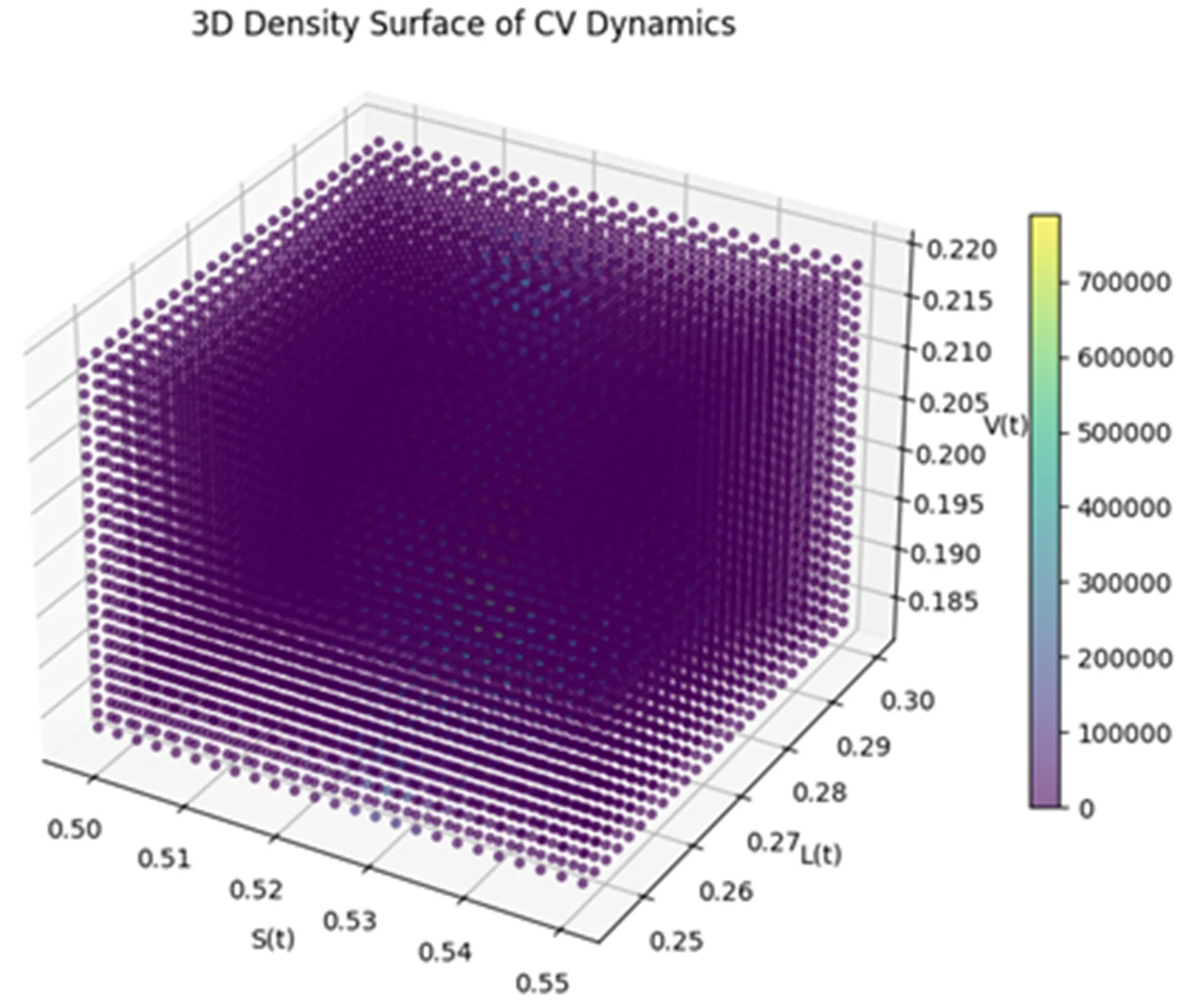
Three-dimensional density representation of the numerical solution (*S*(*t*), *L*(*t*), *V*(*t*)), illustrating the regions visited by the stochastic trajectories.

### 4.3 Separate and combined effects of fractional memory and delay

The fractional-order parameter α and the time-delay parameter τ represent two distinct mechanisms in the proposed model. The fractional order controls the memory structure through the Caputo derivative, whereas τ represents a finite time lag in the infection process. The delay enters the infection term through the delayed states S(t−τ), L(t−τ), and V(t−τ) and through the exponential attenuation factor $e^{-\delta^{\alpha }\tau }$. To distinguish these mechanisms, their effects are examined separately and in combination.

#### 4.3.1 Effect of fractional memory.

First, the effect of fractional memory is investigated by varying α while keeping the delay fixed at τ=0.1. The values considered are


$$\begin{array}{c} \alpha =0.7,\;0.8,\;0.9,\;1.0. \end{array}$$

All other model parameters and numerical settings are kept unchanged. The case α=1 corresponds to the classical first-order model, whereas α<1 introduces fractional-order memory.

Fig 10 illustrates the effect of varying α while the delay remains fixed. The differences among the trajectories reflect the influence of the fractional memory mechanism and are therefore distinct from changes caused by the transmission delay.

**Fig 10:**
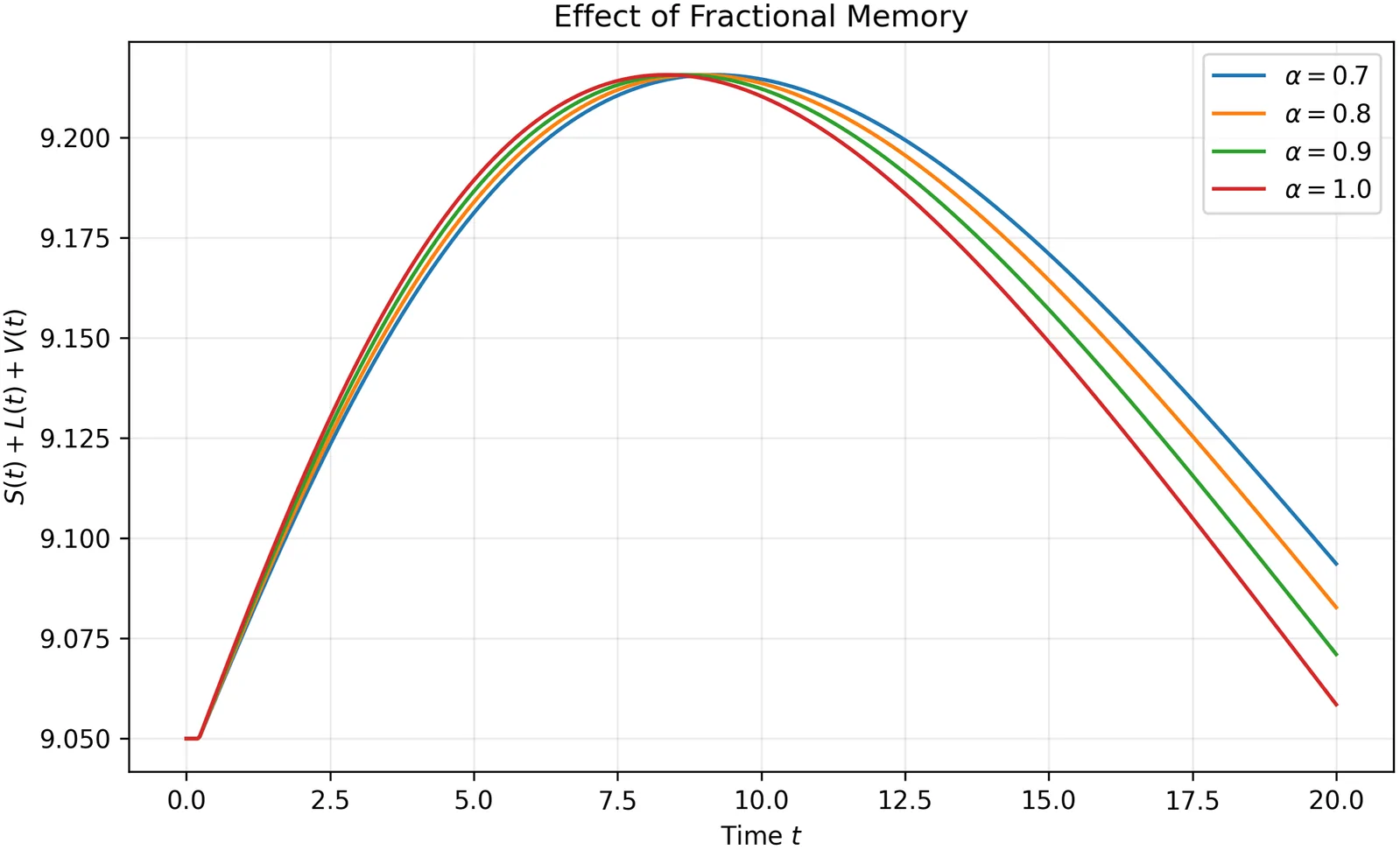
Effect of fractional memory on the computer-virus dynamics for different values of the fractional order α with fixed time delay τ=0.1.

#### 4.3.2 Effect of the transmission delay.

Next, the influence of the delay is examined by fixing the fractional order at α=0.9 and varying


$$\begin{array}{c} \tau =0,\;0.05,\;0.10,\;0.20. \end{array}$$

All other model parameters and numerical settings are kept unchanged. In this experiment, the fractional memory is fixed, so the observed differences are attributed to the delayed transmission mechanism. In particular, variation in τ affects both the delayed states and the exponential attenuation factor $e^{-\delta^{\alpha }\tau }$.

Fig 11 demonstrates the effect of the delayed infection mechanism while the fractional memory is held fixed. The delay changes the timing of the infection input and modifies its effective contribution through the factor $e^{-\delta^{\alpha }\tau }$.

**Fig 11:**
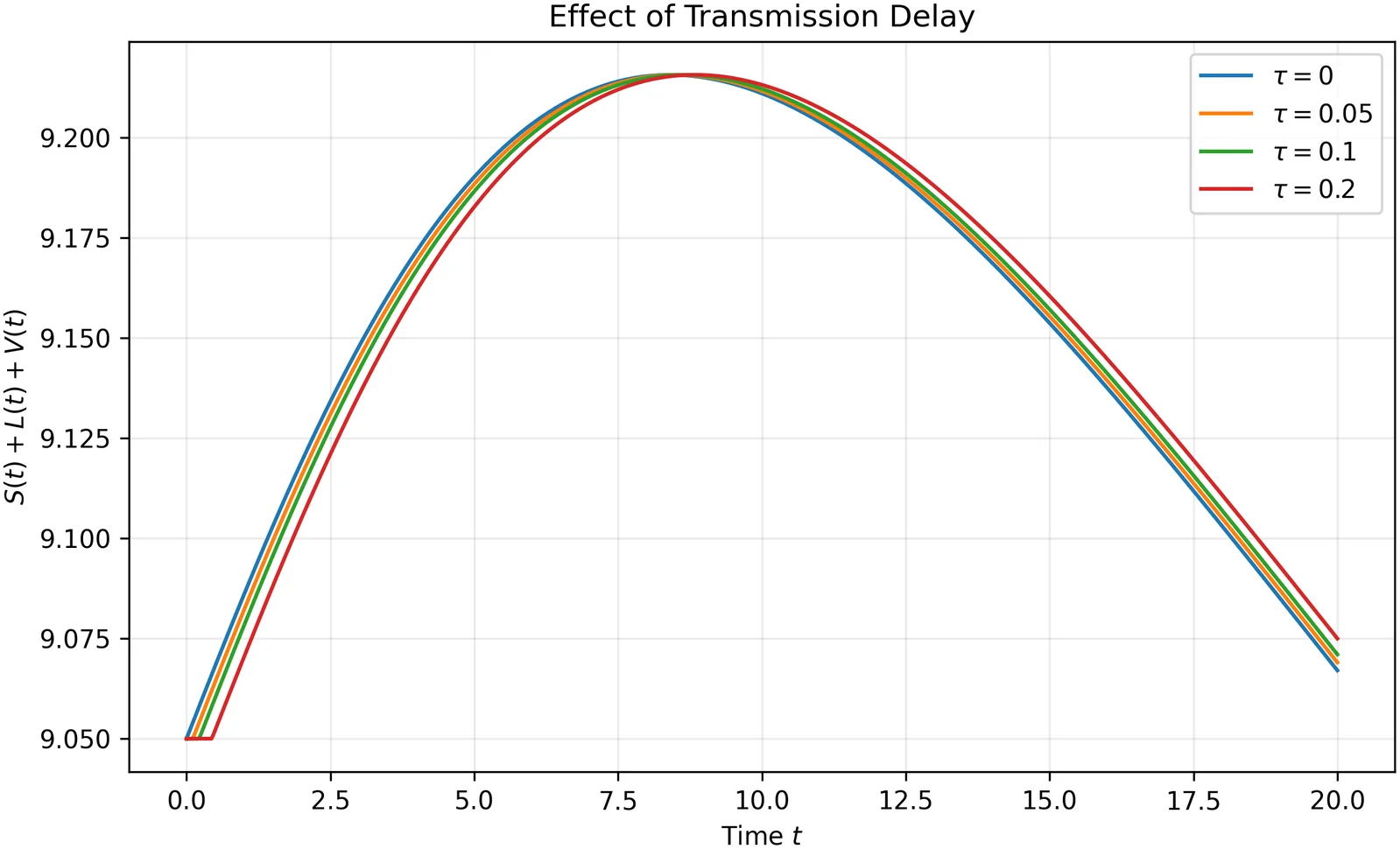
Effect of the transmission delay τ on the computer-virus dynamics for fixed fractional order α=0.9.

#### 4.3.3 Combined effect of fractional memory and delay.

Finally, the combined influence of fractional memory and delay is investigated by varying both α and τ. Representative combinations are considered while all remaining model parameters are kept fixed. This experiment provides a direct assessment of the joint effect of memory and delayed transmission.

Fig 12 illustrates that fractional memory and delay influence the model through different mathematical mechanisms. The fractional order modifies the history dependence of the system, whereas the delay modifies the timing and effective magnitude of the infection input. Their combined consideration therefore provides a more complete representation of the temporal dynamics.

**Fig 12:**
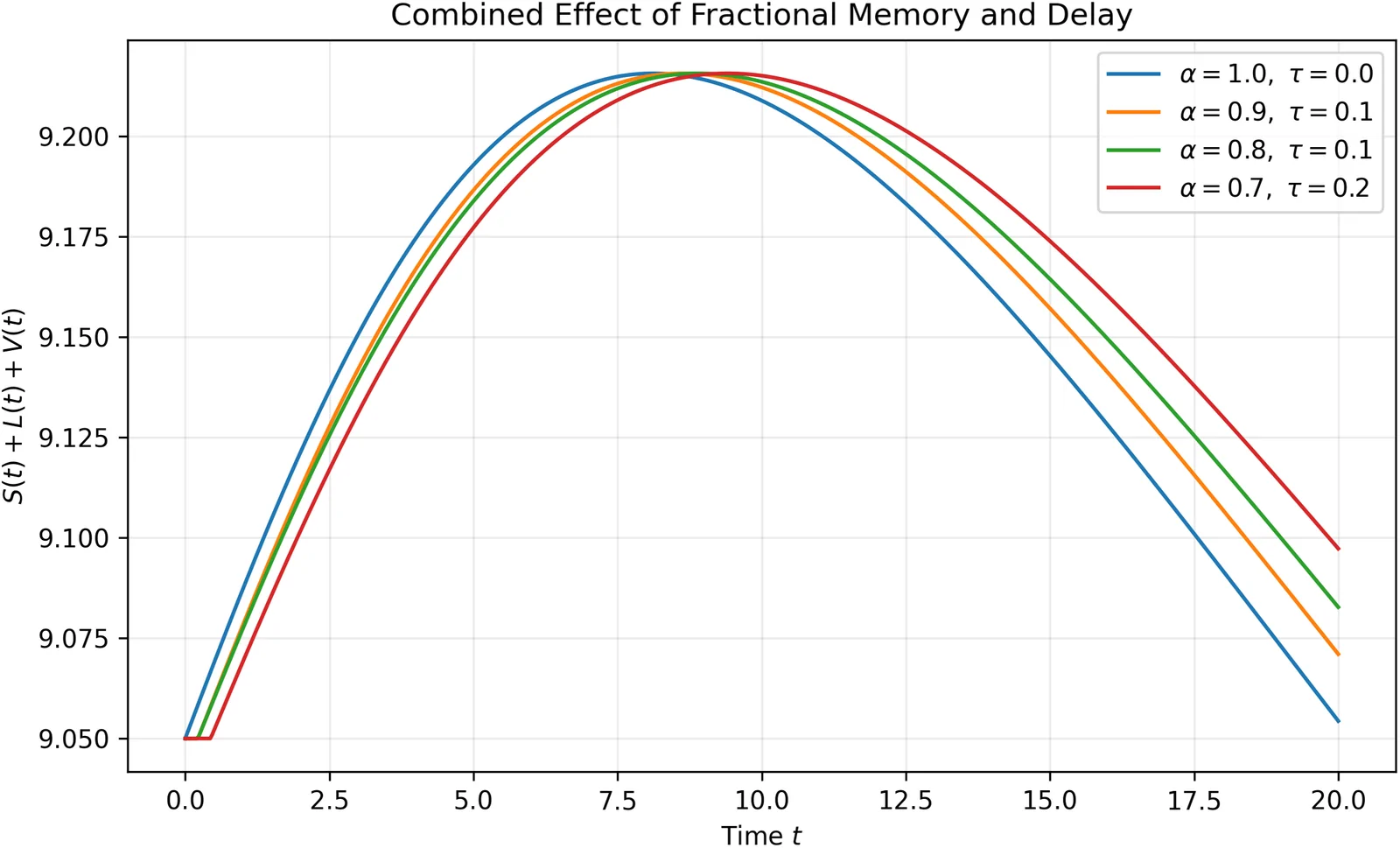
Combined influence of fractional memory and transmission delay on the computer-virus dynamics for different combinations of α and τ.

### 4.4 Influence of stochastic intensity

The influence of stochastic perturbations is investigated by varying the noise intensity while keeping the deterministic parameters, fractional order, delay, initial conditions, history function, and Brownian-motion realization fixed. For simplicity, a common intensity level is considered:


$$\begin{array}{c} \sigma_{1}=\sigma_{2}=\sigma_{3}=\sigma , \end{array}$$

with


$$\begin{array}{c} \sigma =0,\;0.10,\;0.25,\;0.50. \end{array}$$

The case σ=0 corresponds to the deterministic system, whereas positive values introduce stochastic perturbations into the model.

Figs 13–15 show that increasing stochastic intensity produces greater pathwise variability in the computer-virus trajectories. This illustrates the role of random perturbations in the evolution of the uninfected, latent, and seizing computer populations.

**Fig 13:**
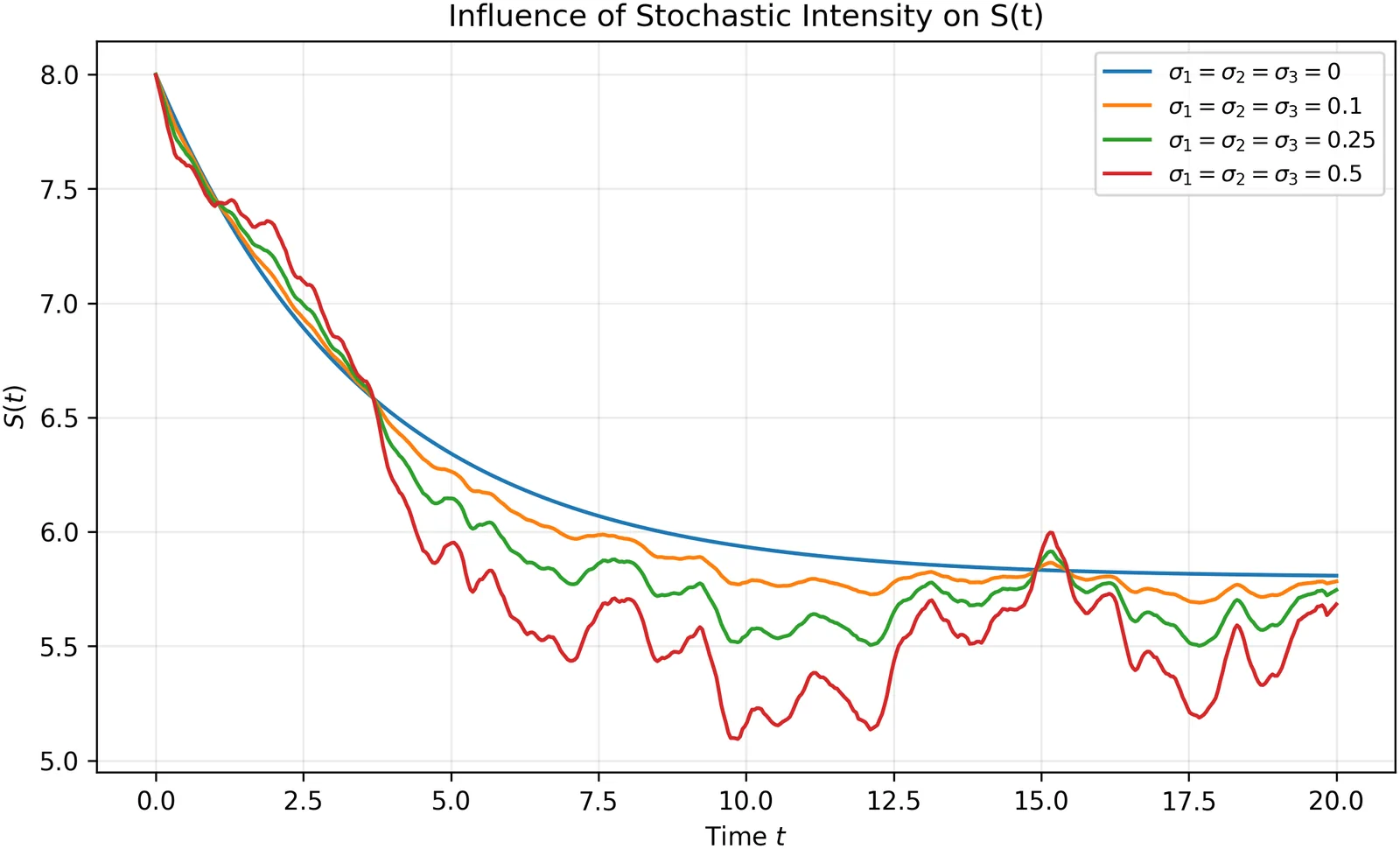
Influence of the stochastic intensity σ on the uninfected computer population *S*(*t*) for $\sigma_{1}=\sigma_{2}=\sigma_{3}=\sigma$.

**Fig 14:**
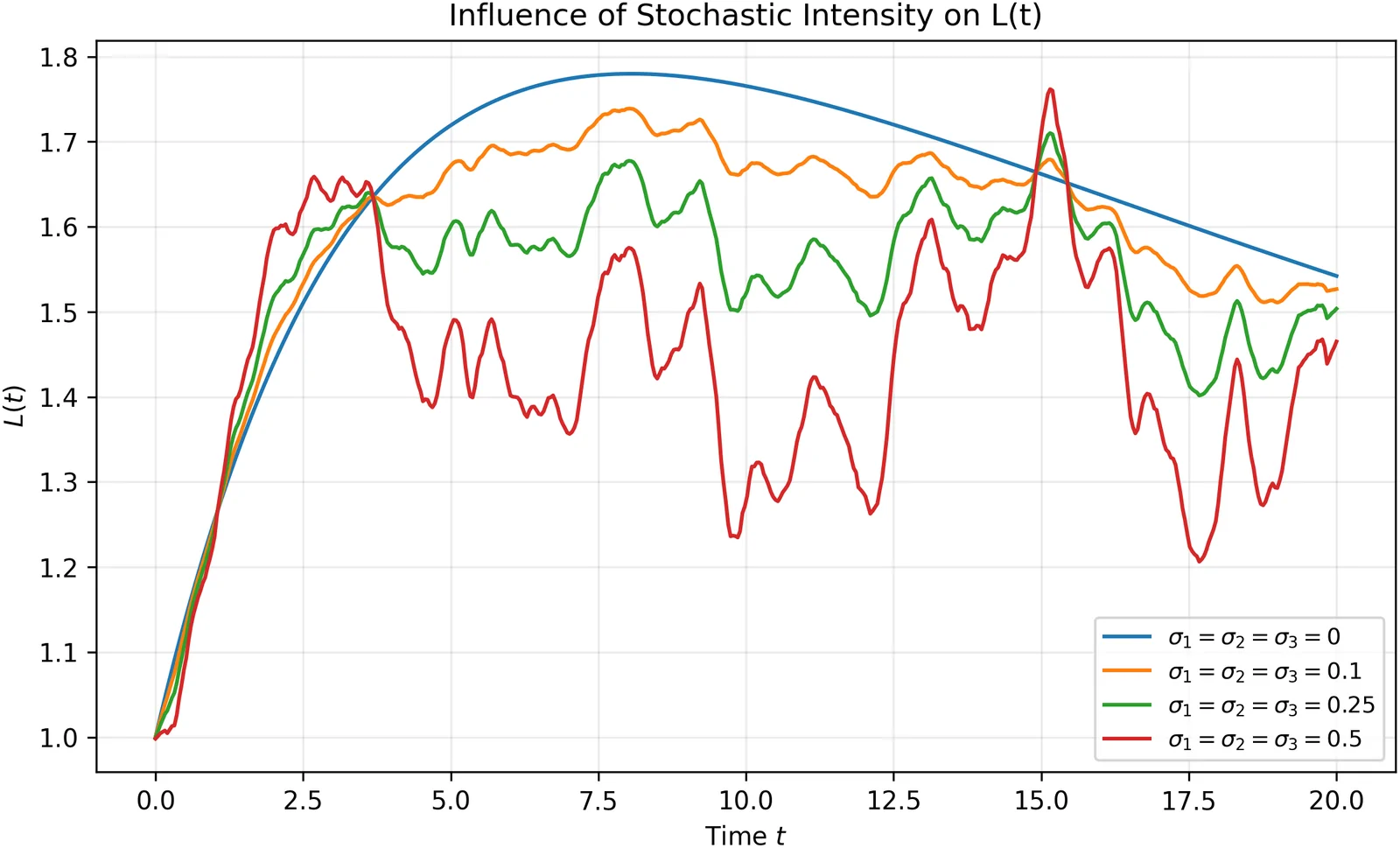
Influence of the stochastic intensity σ on the latent computer population *L*(*t*) for $\sigma_{1}=\sigma_{2}=\sigma_{3}=\sigma$.

**Fig 15:**
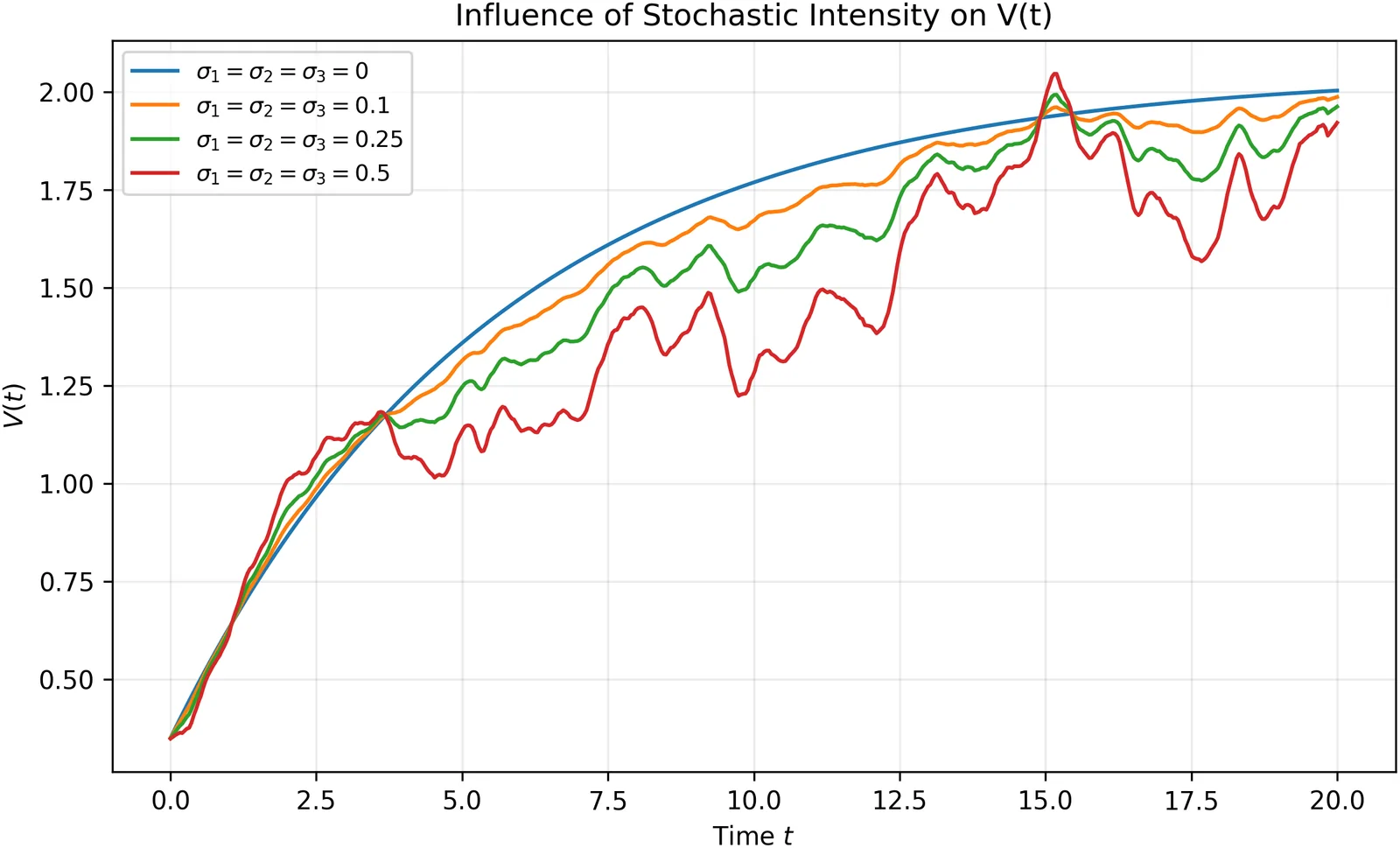
Influence of the stochastic intensity σ on the seizing computer population *V*(*t*) for $\sigma_{1}=\sigma_{2}=\sigma_{3}=\sigma$.

### 4.5 Deterministic and stochastic dynamics

A direct comparison between deterministic and stochastic dynamics is also performed. For the deterministic system,


$$\begin{array}{c} \sigma_{1}=\sigma_{2}=\sigma_{3}=0, \end{array}$$

whereas positive noise intensities are used for the stochastic system. The same deterministic parameters, fractional order, delay, initial and history conditions, time grid, and Brownian-motion realization are used in the comparison.

Fig 16 shows that the deterministic solution follows a smoother trajectory, whereas the stochastic model exhibits fluctuations around the corresponding deterministic behavior. The magnitude of the deviations depends on the selected stochastic intensities and illustrates the contribution of random perturbations to the computer-virus dynamics.

**Fig 16:**
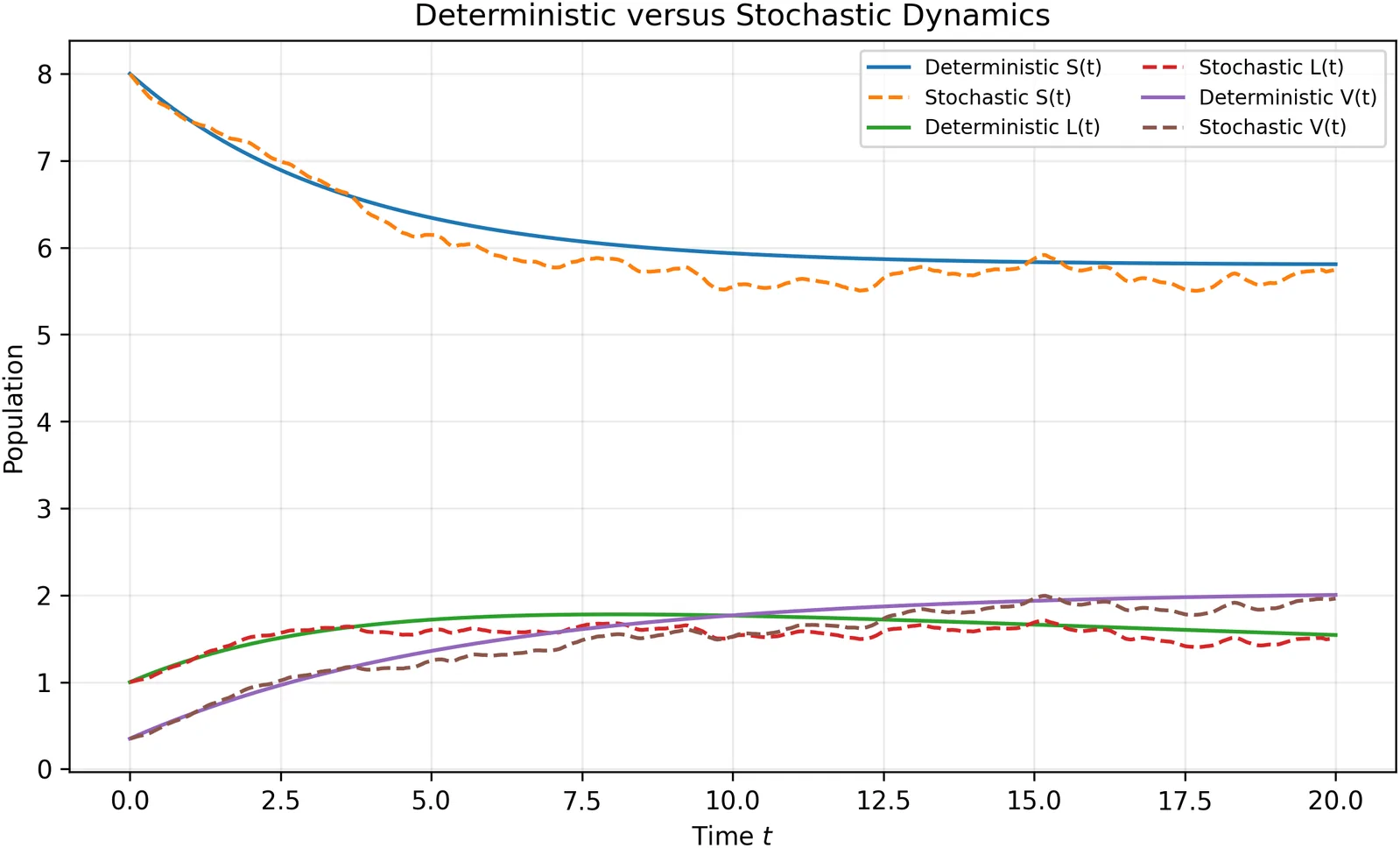
Comparison between deterministic and stochastic dynamics of the computer-virus model. The trajectories of *S*(*t*), *L*(*t*), and *V*(*t*) are shown for the deterministic case $\sigma_{1}=\sigma_{2}=\sigma_{3}=0$ and the stochastic case with positive noise intensities under identical deterministic parameters, fractional order, delay, initial conditions, and history functions.

### 4.6 Comparative numerical simulation

To assess the numerical behavior of the proposed stochastic NSFD scheme, its performance is compared with a fractional Euler–Maruyama (EM) discretization. Both methods are applied to the same stochastic fractional-delay model using identical parameter values, initial and history conditions, fractional order, delay, time-step size, and Brownian-motion realization. This common-path construction provides a consistent pathwise comparison.

Let


$$\begin{array}{c} t_{n}=nh,\;n=0,1,...,N,\;h=\frac{T}{N}, \end{array}$$

and let


$$\begin{array}{c} \Delta B_{n}=B(t_{n+1})-B(t_{n}),\;\Delta B_{n}\sim 𝒩(0,h). \end{array}$$

For $t_{n}-\tau <0$, delayed values are obtained from the prescribed history functions, whereas for $t_{n}-\tau \geq 0$, they are obtained from the numerical solution.

The fractional Euler–Maruyama method is obtained from the fractional integral formulation by discretizing the deterministic convolution terms together with the stochastic contributions. The proposed stochastic NSFD method uses the same model and stochastic forcing but employs a nonstandard discretization designed to provide controlled numerical behavior while preserving the relevant qualitative structure of the model.

Fig 17 shows that increasing the time-step size has a more pronounced effect on the numerical error of the Euler–Maruyama method, whereas the NSFD method maintains comparatively controlled numerical behavior over the tested range. This experiment is intended to assess time-step sensitivity rather than convergence under step-size refinement.

**Fig 17:**
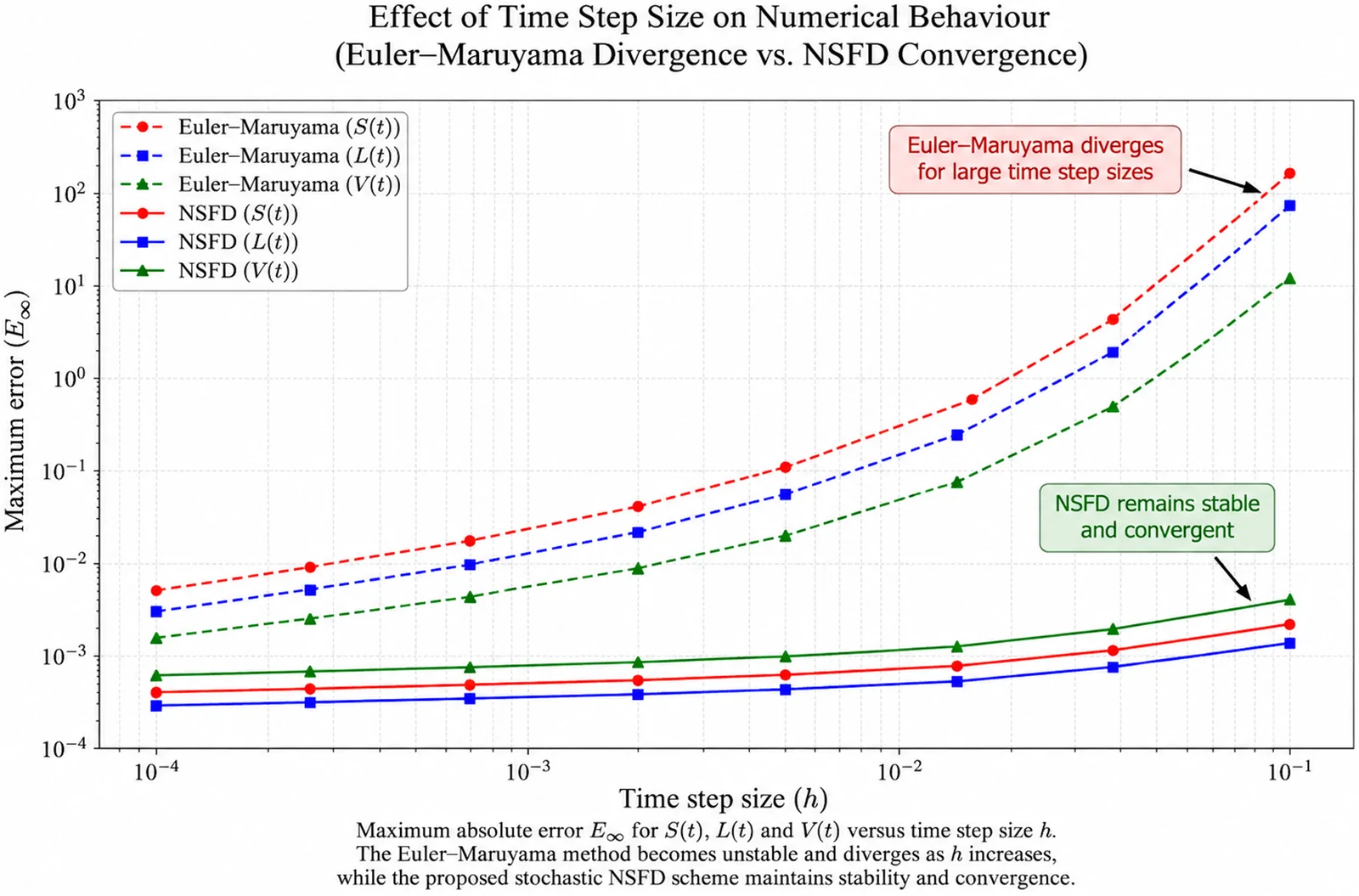
Effect of the time-step size *h* on the numerical behavior of the fractional Euler–Maruyama and stochastic NSFD schemes. The maximum absolute error $E_{\infty }$ is shown for the numerical solutions. The Euler–Maruyama method exhibits a stronger increase in error for larger time steps, whereas the stochastic NSFD scheme maintains comparatively controlled errors over the tested range.

Fig 18 provides a direct visual comparison of the two numerical discretizations. The two methods produce qualitatively comparable trajectories, while their numerical differences are quantified using the maximum error and RMSE below.

**Fig 18:**
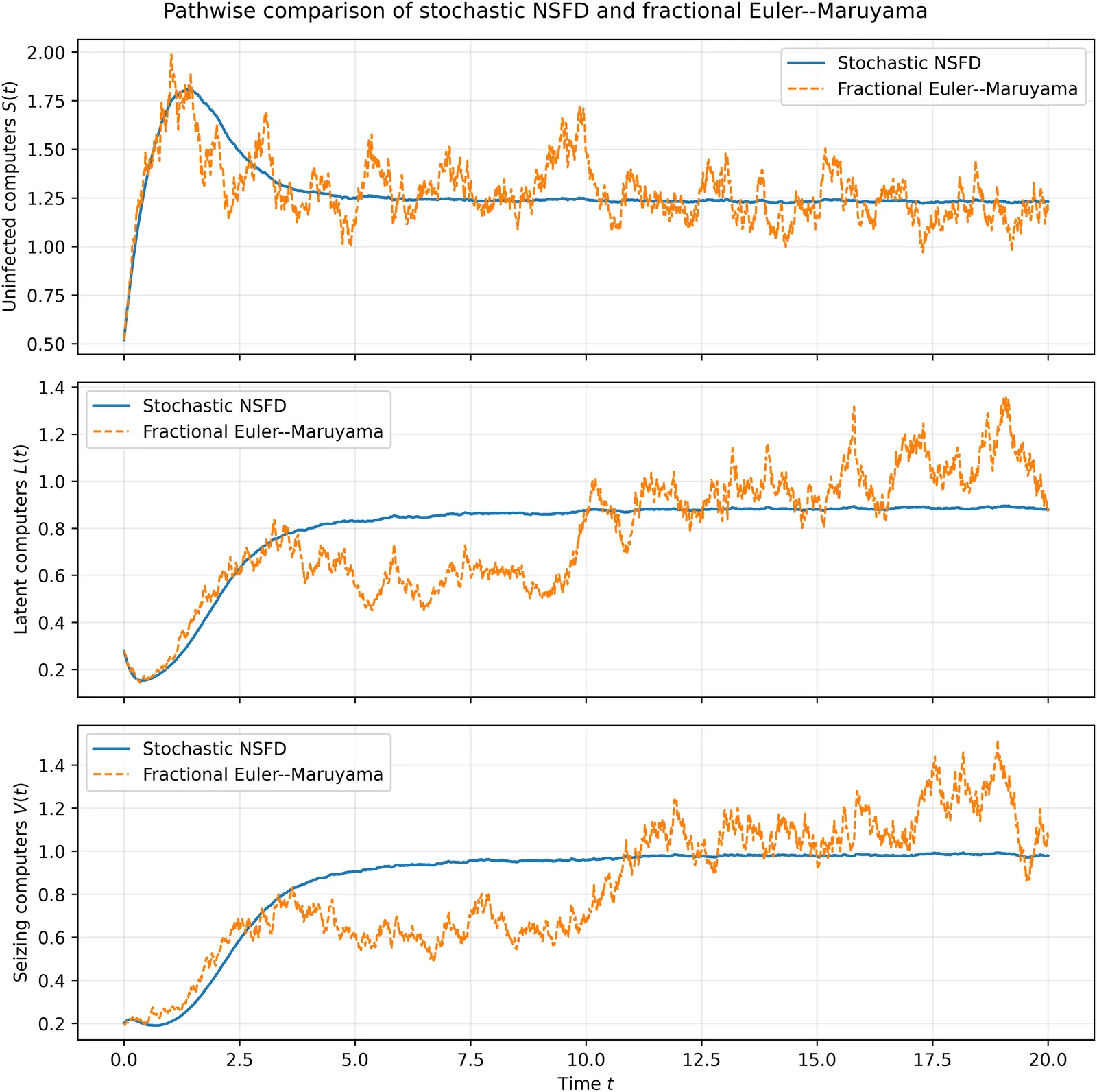
Pathwise comparison of the stochastic NSFD scheme and the fractional Euler–Maruyama method for the uninfected, latent, and seizing computer populations under identical parameter values, initial and history conditions, time-step size, delay, fractional order, and Brownian-motion realization.

### 4.7 Quantitative error comparison

To quantify the numerical discrepancies, a sufficiently refined numerical solution is used as a reference solution,


$$\begin{array}{c} X_{n}^{ref}={\left(S_{n}^{ref},L_{n}^{ref},V_{n}^{ref}\right)}^{T}. \end{array}$$

The reference solution is computed using the same model parameters, initial and history conditions, and Brownian-motion realization, but with a substantially smaller time step (Table 4). For a numerical method M∈{NSFD,EM}, the maximum-norm error is defined by


$$\begin{array}{c} E_{\infty }^{\left(M\right)}=\max_{0\leq n\leq N}{\left\|X_{n}^{\left(M\right)}-X_{n}^{ref}\right\|}_{\infty }, \end{array}$$

where


$$\begin{array}{c} \|X{\|}_{\infty }=\max\{|S|,|L|,|V|\}. \end{array}$$

**Table 4:** Quantitative comparison of the stochastic NSFD and fractional Euler–Maruyama methods with respect to the refined reference solution.

Numerical method	$E_{\infty }$	RMSE
Fractional Euler–Maruyama	0.311015	0.043585
Stochastic NSFD	0.338176	0.047877

The root-mean-square error is defined by


$$\begin{array}{c} {\mathrm{RMSE}}^{\left(M\right)}={\left[\frac{1}{N+1}\sum_{n=0}^{N}{\left\|X_{n}^{\left(M\right)}-X_{n}^{ref}\right\|}_{2}^{2}\right]}^{1/2}. \end{array}$$

For the particular simulation considered, the fractional Euler–Maruyama method gives slightly smaller values of both $E_{\infty }$ and RMSE than the NSFD method. Therefore, these results do not support a claim that the NSFD method is more accurate according to these two error measures. Rather, the numerical advantage examined for the NSFD formulation concerns its structure-preserving construction and comparatively controlled behavior under changes in the time-step size.

### 4.8 Numerical positivity and boundedness

The numerical trajectories were also examined with respect to nonnegativity and boundedness (Table 5). In particular, the conditions


$$\begin{array}{c} S_{n}\geq 0,\;L_{n}\geq 0,\;V_{n}\geq 0 \end{array}$$

were checked throughout the simulation interval, together with the total computer population


$$\begin{array}{c} N_{n}=S_{n}+L_{n}+V_{n}. \end{array}$$

**Table 5:** Numerical preservation of nonnegativity and boundedness for the fractional Euler–Maruyama and stochastic NSFD schemes under the specified simulation conditions.

Numerical method	Negative values observed	Bounded trajectory
Fractional Euler–Maruyama	No	Yes
Stochastic NSFD	No	Yes

Under the parameter values, initial and history conditions, time-step size, fractional order, delay, and Brownian-motion realization used in the numerical experiment, neither method produced negative values of $S_{n}$, $L_{n}$, or $V_{n}$. Both numerical trajectories also remained bounded over the simulation interval. These results are numerical observations for the specified simulation setting and are not intended as a general stochastic positivity or boundedness theorem.

### 4.9 Discussion

The numerical experiments show the impact of the three main mechanisms introduced in the proposed model: the memory effect, the delay in transmission, and the stochastic perturbations. Variation in the fractional order modifies the transient behavior through the memory structure of the Caputo derivative, whereas variation in the delay modifies the timing and effective magnitude of the infection input. In the combined memory–delay experiment, these mechanisms operate on separate but interacting aspects of the model.

The stochastic-intensity experiments demonstrate the effect of increasing the noise level on the pathwise variability of the computer populations. The difference between the smooth deterministic evolution and the fluctuating stochastic trajectories is further illustrated with the deterministic–stochastic comparison. These dynamics in the state space are described with complementary description in the phase representation, heatmap and density representation. The fractional Euler–Maruyama and stochastic NSFD methods exhibit similar qualitative dynamics for the same stochastic realization, suggesting that both methods provide similar dynamics. The quantitative error results reveal that the Euler–Maruyama method has slightly smaller errors for the specific experiment analyzed and the time-step sensitivity experiment suggests comparatively controlled behavior for the NSFD scheme for larger time steps tested. In this sense, the proposed NSFD method is primarily motivated by the fact that it is formulated in such a way that the structure is preserved, and that it is numerically robust, and not by the claim of having, in general, lower error than Euler–Maruyama.

Overall, the simulations support the analytical results by providing a pictorial representation of the effects of fractional memory, transmission delay, and stochastic forcing on the computer-virus populations’ temporal evolution. The extra numerical explorations also clearly separate the memory/delay effects and show the importance of the stochastic perturbation in the proposed model.

## 5 Conclusion

The behavior of computer viruses has been studied by developing a stochastic fractional-order model that includes the fundamental computational parameters affecting virus propagation. The uniqueness and positivity and boundedness of the solutions are established theoretically. The two equilibria, namely the computer-virus-free and virus-persistent equilibria, were derived, and the basic reproduction number was computed using the next-generation matrix method. Local and global stability of the equilibrium points were explored and a sensitivity analysis of the parameters that determine the transmission of the viruses was carried out. The numerical simulations demonstrated that the proposed stochastic non-standard finite difference scheme still keeps the positivity of the numerical solution and boundedness under the simulation conditions tested, and also has a stable numerical behavior in comparison with the fractional Euler–Maruyama method. The graphical outputs had been consistent with the theoretical analysis, and they showed the influences of fractional memory, transmission delay and random fluctuation on computer-virus dynamics. The results from a practical cybersecurity point of view suggest that prevention of virus transmission and development of better virus detection, removal, and recovery processes are important. The primary drawback of this study is the parameter values chosen are not a complete real-world time-series calibration, and the stochastic stability properties are mainly explored numerically. In the future the data-driven parameter estimation and validation will be the focus of future research together with rigorous stochastic stability and convergence analysis of the proposed numerical scheme. Other extensions may include network heterogeneity, more realistic cyber security interventions, and optimal control strategies. In conclusion, the study highlights the effectiveness of the stochastic fractional-order model and the structure-preserving numerical simulation in understanding and analyzing the complex computer-virus dynamics.
